# Whole genome sequencing reveals possible host species adaptation of *Streptococcusdysgalactiae*

**DOI:** 10.1038/s41598-021-96710-z

**Published:** 2021-08-30

**Authors:** Davide Porcellato, Marit Smistad, Siv Borghild Skeie, Hannah Joan Jørgensen, Lars Austbø, Oddvar Oppegaard

**Affiliations:** 1 Faculty of Chemistry, Biotechnology and Food Science, The Norwegian University of Life Sciences, P.O. Box 5003, 1432 Ås, Norway; 2 Norwegian Veterinary Institute, PB 750 Sentrum, 0106 Oslo, Norway; 3 TINE SA, P.O. Box 7, 0901 Oslo, Norway; 4 Department of Medicine, Haukeland University Hospital, 5021 Bergen, Norway

**Keywords:** Bacterial genomics, Bacterial genes, Pathogens

## Abstract

*Streptococcusdysgalactiae* (SD) is an emerging pathogen in human and veterinary medicine, and is associated with several host species, disease phenotypes and virulence mechanisms. SD has traditionally been divided into the subspecies *dysgalactiae* (SDSD) and subsp*.* *equisimilis* (SDSE), but recent molecular studies have indicated that the phylogenetic relationships are more complex. Moreover, the genetic basis for the niche versatility of SD has not been extensively investigated. To expand the knowledge about virulence factors, phylogenetic relationships and host-adaptation strategies of SD, we analyzed 78 SDSD genomes from cows and sheep, and 78 SDSE genomes from other host species. Sixty SDSD and 40 SDSE genomes were newly sequenced in this study. Phylogenetic analysis supported SDSD as a distinct taxonomic entity, presenting a mean value of the average nucleotide identity of 99%. Bovine and ovine associated SDSD isolates clustered separately on pangenome analysis, but no single gene or genetic region was uniquely associated with host species. In contrast, SDSE isolates were more heterogenous and could be delineated in accordance with host. Although phylogenetic clustering suggestive of cross species transmission was observed, we predominantly detected a host restricted distribution of the SD-lineages. Furthermore, lineage specific virulence factors were detected, several of them located in proximity to hotspots for integration of mobile genetic elements. Our study indicates that SD has evolved to adapt to several different host species and infers a potential role of horizontal genetic transfer in niche specialization.

## Introduction

*Streptococcusdysgalactiae* (SD) is a potent pathogen capable of producing a wide spectrum of clinical manifestations and infecting a broad range of host species. Based on DNA-relatedness and phenotypic characteristics, SD is divided into *Streptococcus* *dysgalactiae* subspecies *dysgalactiae* (SDSD) and subspecies *equisimilis* (SDSE)^1^. SDSD are alpha-haemolytic or non-haemolytic strains belonging to Lancefield group C that are mainly associated with animals, while SDSE are beta-haemolytic strains belonging to Lancefield groups A,C, G or L, and cause miscellaneous infections in humans and domestic animals^1^.

SDSD is reported as an important pathogen in meat sheep and dairy cows. The pathogenesis in the two host species is remarkably different. In sheep flocks, SDSD-infections are associated with outbreaks of septic arthritis in lambs less than four weeks old, whilst in bovine dairy herds SDSD is a frequent cause of mastitis.

In Norway, the relative importance of SDSD-infections in livestock has increased over the last decade. In a survey from 2018, 5.6% of 1700 Norwegian sheep farms had experienced outbreaks of infectious arthritis in lambs^2^. At the same time, the prevalence of SDSD intramammary infections is increasing in bovine dairy herds, and SDSD is now the third most common cause of clinical mastitis in dairy cows in Norway^3^. The two industries currently define streptococcal mastitis in dairy cows and streptococcal joint infections in lambs to be among the major challenges in Norwegian livestock production, because of their negative effects on animal health and welfare, production and antibiotic usage.

In the past decades, SDSE has emerged as an important human pathogen. Traditionally, SDSE in humans has been regarded as a potentially zoonotic pathogen, but recent phylogenetic studies based on multilocus sequence typing (MLST) have suggested distinct host-adapted subpopulations of SDSE^4,5^.

Although several studies have involved whole genome sequencing of SDSE, very few studies have sequenced SDSD^6,7^. Genomic investigations of SDSD to reveal factors associated with virulence, persistence in the environment and host specificity can contribute to enhancing our understanding of pathogenicity and transmission. The purpose of this study was to explore by whole genome sequencing the diversity of SDSD from sheep and cattle in Norway, and to identify genetic factors that might contribute to host adaptation. Furthermore, a comparative genome analysis was performed between bovine- and ovine associated SDSD, and SDSE from various other host species.

## Results

### Genome statistics of SDSD

In this study, we sequenced 60 new genomes of SD, comprising 37 and 23 isolates from cows and sheep, respectively (Table S1). All the isolates were SDSD with the exception of one ovine isolate which was classified as SDSE. The genomes of an additional 18 SDSD of bovine origin were retrieved from public databases and from Velez et al.^6^ and included in the analysis.

The genome size of the 78 SD isolates from cows and sheep had an average of 2.04 MB (2.04 ± 0.1 for bovine isolates and 2.02 ± 0.05 for ovine isolates), an average number of CDS of 1990 (1993 ± 95 in bovine isolates and 1992 ± 40 in ovine isolates).

### Virulence factors of SDSD

The genomes of all SDSD isolates of bovine and ovine origin were equipped with numerous virulence genes (Table 1). Several were found to be ubiquitous, including genes involved in adhesion (*fnbA*, *fnbB*, *gapC* and surface enolase), immune evasion (a spyCEP homolog) and dissemination (*padA* and a DNAseB homolog). The adhesin *demA* has previously been characterized in SDSD and was detected in 31 of the 78 SDSD isolates included in this study. Immunoglobulin-binding virulence factors were identified in all genomes, where 48 isolates contained the macroglobulin and immunoglobulin binding protein MIG, and 29 harbored the macroglobulin, albumin and immunoglobulin binding protein MAG.

**Table 1:** Virulence factors of *Streptococcus* *dysgalactiae* subsp. *dysgalactiae* (SDSD) and *Streptococcus* *dysgalactiae* subsp. *equisimilis* (SDSE)*.*

	Virulence factor	GenBank ID	SDSD		SDSE				
			Cow (*n* = 55) (%)	Sheep (*n* = 22) (%)	Human (*n* = 35) (%)	Horse (*n* = 20) (%)	Dog (*n* = 8) (%)	Swine (*n* = 7) (%)	Fish (*n* = 5) (%)
**Adhesin**	*fnbA*	Z22150	100	100	0	0	0	0	0
	*fnbB*	Z22151	100	100	100	100	100	100	100
	*demA*	AJ243529	38	45	0	0	0	0	0
	pilus1		0	0	100	95	100	100	trunc
	pilus2		0	0	86	25	0	0	0
	*gfba/prtf1*	U31115	0	0	39	5	100	0	0
	*srr*	EFY01869	100	100	0	0	0	0	0
	*gapC*	X97788	100	100	100	100	100	100	100
	Surface enolase	AAT86712	100	100	100	100	100	100	100
	*lmb1*	AB040535	0	0	100	0	0	0	0
	*lmb2*	SUN51641	100	100	0	0	0	0	0
	Streptlolysin O	AE004092	0	0	100	0	0	0	0
**Toxin**	NAD	AAK33265	0	0	100	0	0	0	0
	Streptolysin S	AF067649	0	0	100	100	100	100	100
	Sil	KF188416	2	0	64	25	0	100	0
	c5a-peptidase	J05229	0	0	100	0	0	0	0
	spyCEP-like	SUN48961	100	100	trunc	trunc	100	100	100
**Immune evasion**	proteinG	Y00428	0	0	69	0	0	0	0
	MIG	Z29666	65	55	0	0	0	0	0
	MAG	L27798	35	45	0	0	0	29	0
	*drsG*	AB508817	0	0	42	0	0	0	0
	DNAseB-like	NP_269989	100	100	0	0	0	0	0
	mf2*	NP_268944	11	4	0	0	0	0	0
	mf3*	NP_269520	38	87	8	0	0	0	0
	mf4*	AAM79702	0	17	0	20	0	0	0
	sdn*	AAM80016	4	4	8	5	0	57	0
	sda2*	WP002988811	7	30	17	0	0	0	0
**Spread**	Streptokinase	K02986	0	0	100	0	0	0	0
	*padA*	AJ441115	100	100	0	0	0	0	0
	*skc*_horse	AF104301	0	0	0	100	0	0	0
	*skc*_pig	AF104300	0	0	0	0	0	100	0
	Putative skc	WP129556387	0	0	0	0	0	15	100
	Putative skc	VTS33028	0	0	0	0	100	0	0
	speC*	AAK33664	9	4	0	0	0	0	0
	*speG*	AF124499	0	0	56	20	100	29	80
**Superantigen**	speK*	WP011054728	11	4	0	0	0	0	0
	speL*	WP011017837	5	0	0	0	0	0	0
	speM*	WP011017838	5	0	0	0	0	0	0

Data presented as percentage of isolates harboring the virulence factor. The four isolates that likely represent cross species transmission have been omitted from the table.

*Trunc* truncated gene, *skc* streptokinase C. * denotes phage associated genes.

No isolates harbored superantigens *speA*, *speH*, *speI*, *speJ*, *speQ*, *speR*, *ssa* or *smeZ.*

**Figure 1:**
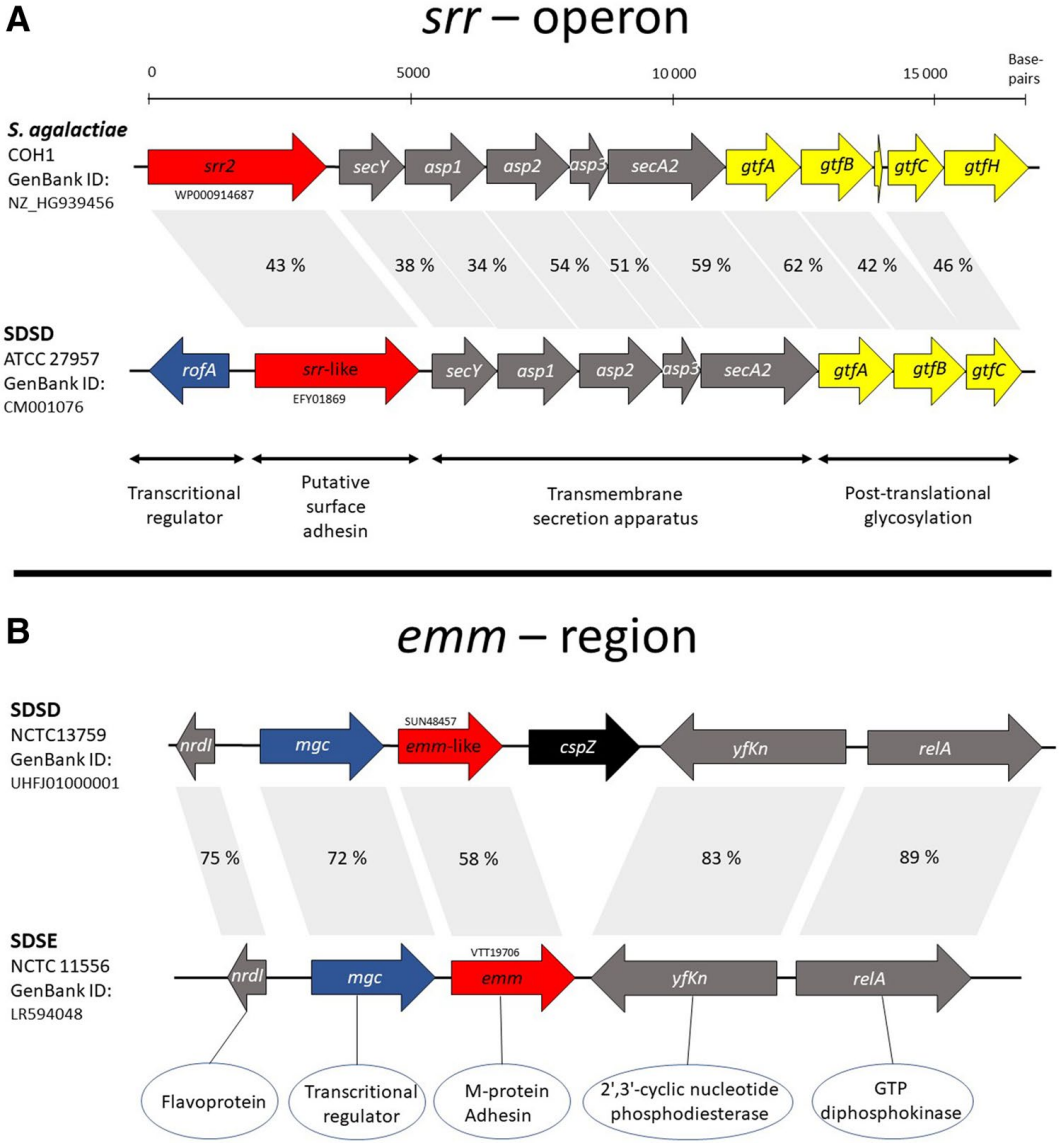
Genetic organization of the Srr-locus and *emm*—region in *Streptococcus* *dysgalactiae.* Panel A depicts a comparison of the novel *srr*-like locus detected in SDSD to the *srr*-operon of *S.* *agalactiae*. Two distinct *srr* variants have been described in *S.* *agalactiae*, denoted *srr1* and *srr2*. The *srr*-like gene and *rof*A transcriptional regulator in SDSD is more similar to *srr1* of *S.* *agalactiae*. However, the overall genetic organization of the SDSD *srr*-like operon resembles the *srr2*-locus, and is thus presented in the figure. The functional descriptions are inferred from the characterization of the *srr*-locus in *S.* *agalactiae* (ref Mistou (ref 10)). Panel B shows an alignment of the *emm*—region in SDSD and in human associated SDSE isolates. SDSD genomes harbour an additional gene, *cspZ*, predicted to encode a cell wall surface protein.

Notably, pilus-operons were absent from all the SDSD isolates. However, at the genomic location of pilus island 1, they harbored a serine-rich repeat glycoprotein-operon resembling the fibrinogen-binding Srr-locus previously characterized in *Streptococcus* *agalactiae* (Fig. 1A). In addition to the *srr*-like gene, the operon includes a transcriptional regulator, a SecA2 protein-transport apparatus and three genes putatively involved in Srr-glycosylation, *gtfA*, *gtfB* and *gtfC* (Fig. 1A). Although the sequence homology to the *S.* *agalactiae* Srr-operon was limited to ~ 50%, the functional domains were conserved.

**Figure 2:**
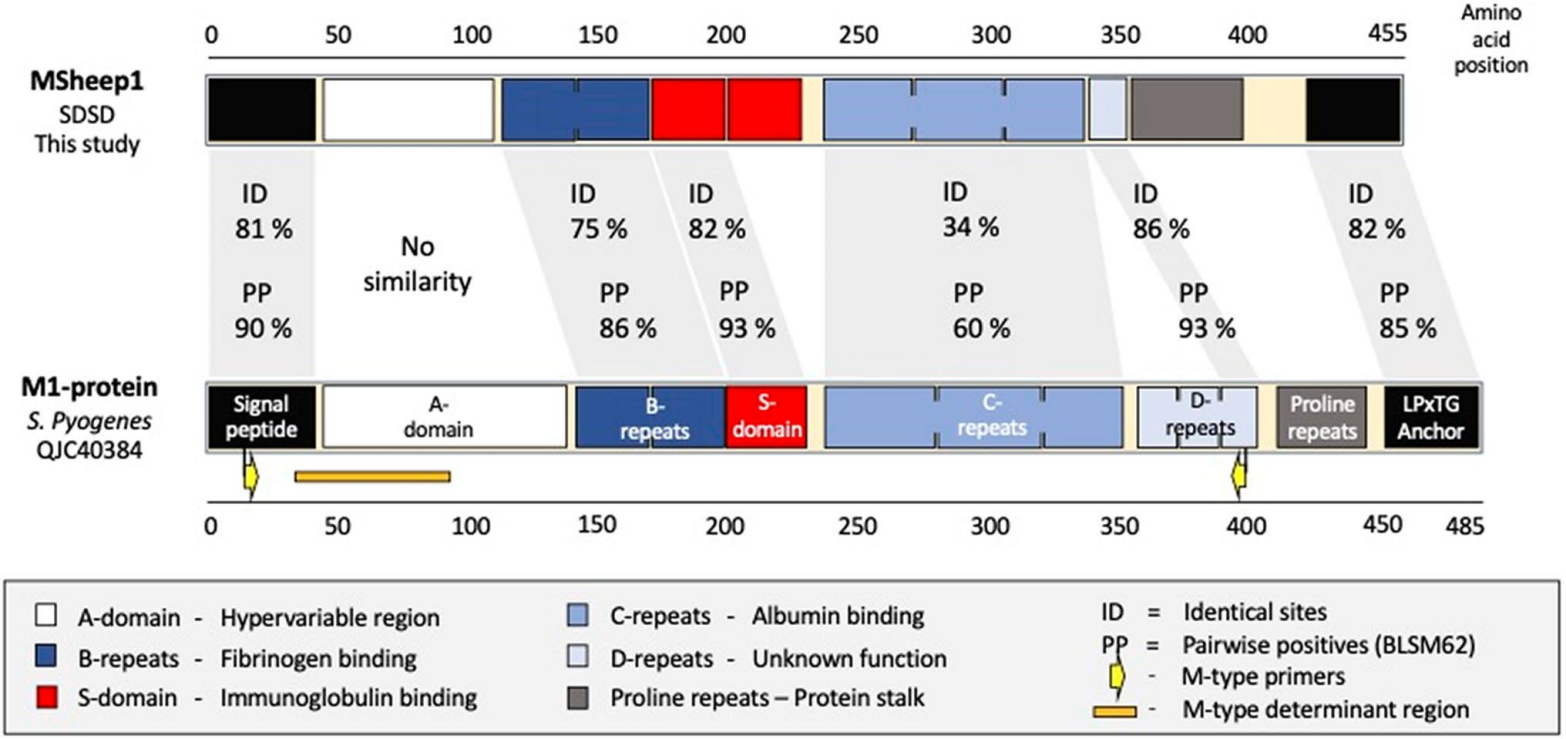
Structure of the M-protein identified in *Streptococcus* *dysgalactiae* subsp. *dysgalactiae* and in *Streptococcus* *pyogenes.* Global alignment of the M1-protein of *S.* *pyogenes* and one of the M-like proteins detected in SDSD in this study. The comparison was performed using Geneious alignment with the BLOSUM62 cost matrix. Sequence similarity is presented as identical sites (ID) and pairwise positives (PP). The proline repeat region functions as a stalk that protrudes the active domains from the bacterial cell surface. It consists of very short proline rich repeats of 3–5 amino acids of variable quantity, making sequence alignment less informative.

**Figure 3:**
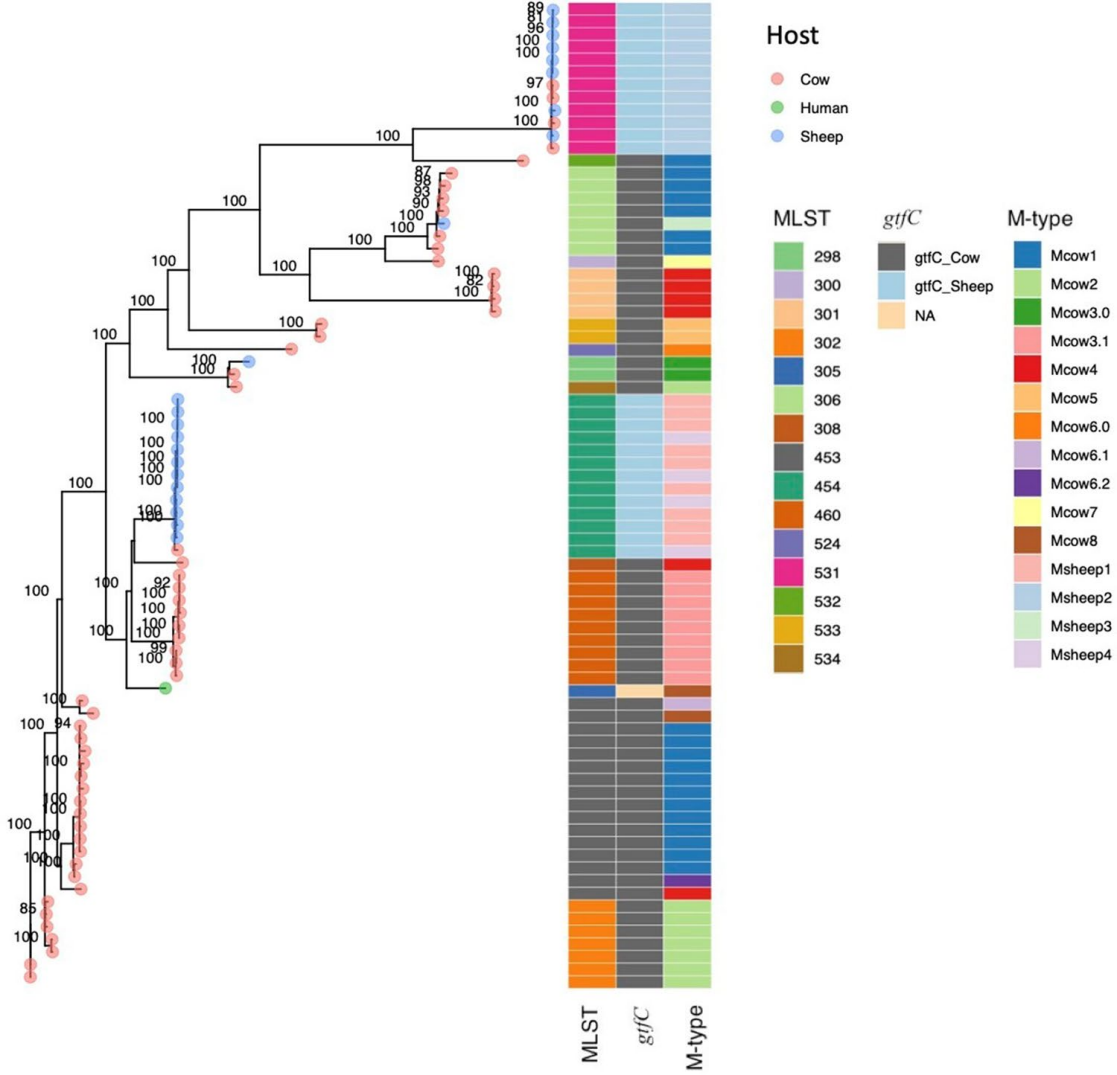
Phylogenetic tree of *Streptococcus* *dysgalactiae* subsp. *dysgalactiae* isolates obtained from the alignment of 752 single orthologue genes and information about MLST, gtfC-type and M-type. The numbers above the branches are support values from 100 bootstrap replicates.

The most important virulence factor and molecular typing tool of SDSE and *S.* *pyogenes*, the M-protein, has not previously been identified in SDSD. Interestingly, we located an *emm*-like gene in a genetic context resembling that of the *emm*-gene in SDSE: downstream from *nrdI* and an mgc-regulator, and upstream from 2,3 phosphodiesterase and *relA* (Fig. 1B). The homology is also striking on a protein-level. These M-like proteins have a predicted coiled structure, contain repetitive elements at the C-terminal end, and harbor a YSIRK-signal-peptide and a transmembrane LPxTG anchor with very high homology to SDSE and *S.* *pyogenes* M-proteins (Fig. 2). The *emm*-typing PCR primers recommended by CDC have 4 mismatches in the forward primer and 1 mismatch in the reverse primer when aligned to the SDSD *emm*-like gene, which likely explains why this subspecies appears to be non-typable using this protocol. Applying the CDC *emm*-typing scheme in silico we categorized the SDSD-genomes into different *emm*-types for phylogenetic purposes (Fig. 3, Table S1). At the recommendation of the curators these new *emm*-like genes have not been deposited in the *emm*-database.

### MLST and phylogenetic analysis of SDSD

Molecular typing revealed 14 different MLST-profiles among the SDSD isolates, including 5 novel profiles (Table S1, Fig. 3). Isolates from dairy cows displayed 13 different MLST-types, of which the majority have previously been reported in association with bovine mastitis, while isolates from sheep were more homogenous and grouped into four STs. Two of the twenty-three isolates of ovine origin had an MLST-profile identical to SDSD previously associated with bovine mastitis.

Phylogenetic analysis was reconstructed from 752 gene clusters that were identified as single orthologue genes by the pangenome analysis of all the isolates included in the study. The majority of ovine isolates clustered within 2 main clades (Fig. 3). Pairwise average nucleotide identity was larger than 98% between all the isolates of SDSD from cows, sheep and the human isolate.

### Dissection of host specific traits in SDSD

Whole genome comparison of isolates derived from bovine and ovine hosts was performed to identify potential host specific signatures. However, the SDSD genomes were highly homogenous, and no single gene or genetic regions were found to be uniquely associated with host species. Searching for genes displaying < 90% similarity between isolates of bovine and ovine origin we identified several surface exposed virulence factors with high genetic variability. However, these genes also displayed substantial heterogeneity within each host-group, and the allelic variants generally corresponded with the MLST-profile.

Interestingly, the glycosylation gene *gtfC* of the putative *srr*-operon existed in two distinct allelic variants displaying 88% similarity. The distribution of these two variants was highly concordant with origin; 50 of the 55 bovine isolates harbored allele A, while 20 of the 22 ovine SDSD isolates contained allelic variant B (Fig. 3). The ovine isolate identified as SDSE did not harbor an *srr*-operon.

### Comparison of SDSD and SDSE

**Figure 4:**
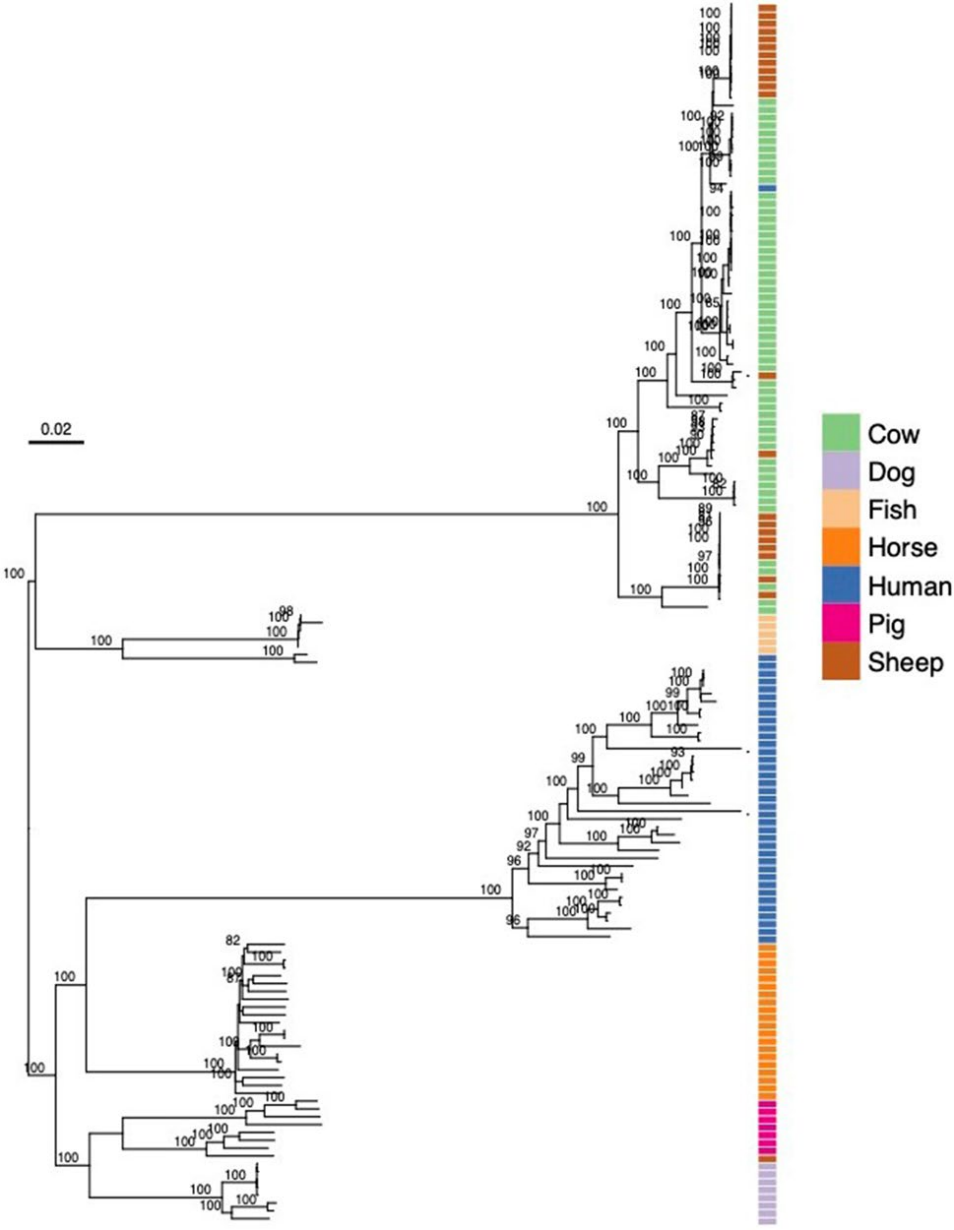
Midpoint-rooted phylogenetic tree of *Streptococcus* *dysgalactiae* subsp. *dysgalactiae* (n = 78) and *Streptococcus* *dysgalactiae* subsp. *equisimilis* (n = 78) included in the study. Colours on the side of each plot indicate the origin of the isolate. Scale indicates substitutions per site. The numbers above the branches are support values from 100 bootstrap replicates.

The genomes of 77 SDSE from different host species (human, pig, fish, dog and horse) and one isolate of SDSD of human origin were added to the analysis for comparative purposes (Table S1). These included 40 newly sequenced genomes isolated from human, dog, horse and pig (24, 6, 4 and 6, respectively). In total, 78 SDSD and 78 SDSE were used for phylogenetic analysis (reconstructed from the 752 gene clusters described above). The phylogenetic analysis showed a clear separation of the two subspecies and of isolates from different host species (Fig. 4). In addition, one isolate of human origin clustered with the SDSD clade. This isolate was obtained from a man suffering from prosthetic valve endocarditis, without verified exposure to livestock^8^.

**Figure 5:**
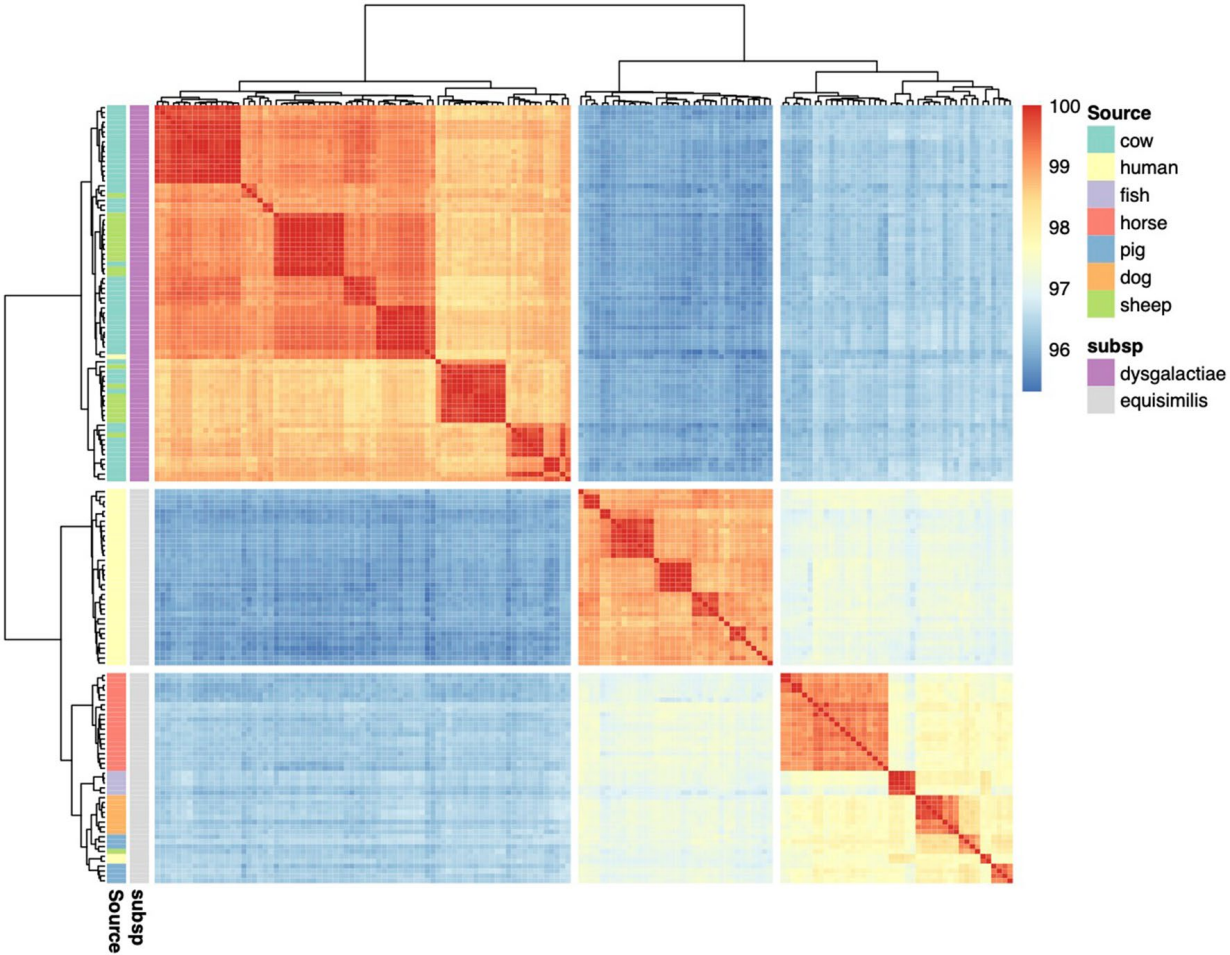
Heatmap of the average nucleotide identity between the whole genome sequences of both subspecies of *Streptococcus* *dysgalactiae* (n = 156)*.*

**Figure 6:**
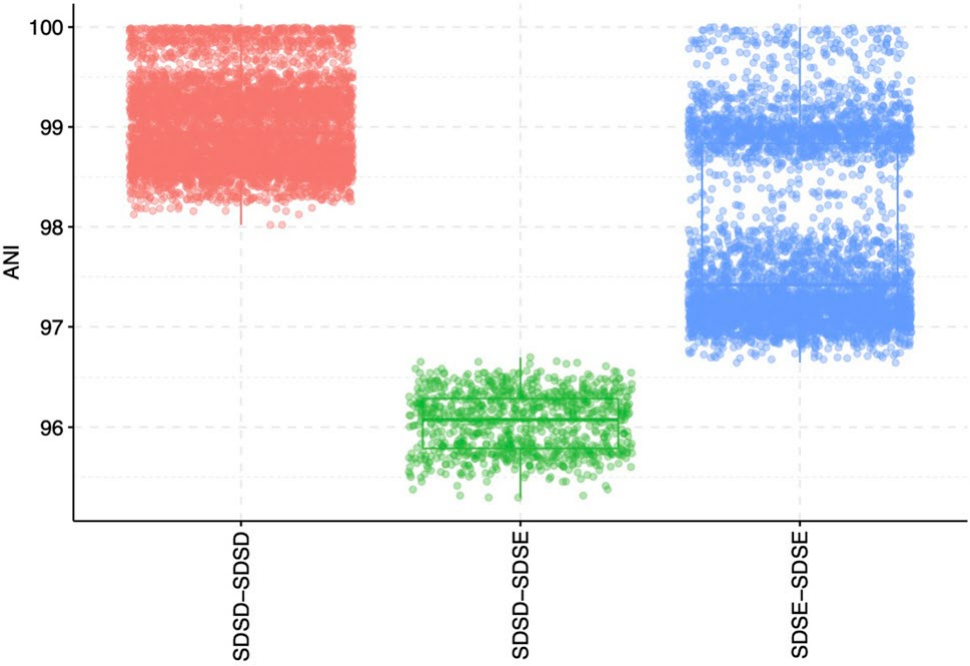
Distribution of pairwise average nucleotide identity within and between the two subspecies of *Streptococcus* *dysgalactiae.* SDSD: *S.* *dysgalactiae* subsp. *dysgalactiae,* SDSE: *S.* *dysgalactiae* subsp. *equisimilis.*

The fastANI algorithm was employed for pairwise comparison of all the genomes. Similar to the phylogenetic analysis, ANI values clearly separated the SD isolates into several clades with distinct delineation of the two subspecies (Fig. 5). The SDSE isolates could be further divided based on the host species. One group contained all the human isolates, except two isolates where the source of infection was suspected to be fish (DB49998-05 and DB60705-15). The second SDSE group contained all the animal isolates, but this group was clearly further separated by host. One isolate from a sheep was identified as SDSE and clustered together with isolates in the pig clade. The average ANI values for the pairwise comparison within the two subsp. were 99.0 and 97.9% for SDSD and SDSE, respectively (Fig. 6). Between the two subsp. the average ANI values were 96.0%. Clear grouping was detected in the ANI value for SDSE, reflecting the two clusters detected by phylogenetic and pangenome analysis.

### Pangenome analysis

Pangenome analysis of the altogether 156 bacterial isolates identified a total of 6464 gene clusters and an estimated pangenome size of 9137 (Chao1 index). Binomial mixture model estimated a pangenome size of 8669 gene clusters and a core-genome of 871 gene clusters (13.5% of total). When considering both subsp. included in the SD species an open pangenomes was detected by Heaps´ law (alpha 0.81). However, clear differences were detected between the two subsp. Isolates of SDSD have a more closed pangenome compared to the SDSE isolates (alpha 0.97 and alpha 0.78, respectively). This also reflects the number of gene clusters identified between the two subsp. (3550 and 5845 for SDSD and SDSE, respectively).

### Virulence profiling and host adaptation

All the isolates were screened for presence of virulence factors (Table 1). The adhesins FnbA, DemA and the new putative Srr-glycoprotein were found to be unique for SDSD. Pilus islands and Streptolysin S were restricted to SDSE isolates. Sub specialization within SDSE was observed, and C5a-peptidase, drsG and the toxins streptolysin O and NAD were exclusively detected in human associated SDSE. Moreover, the distribution of various host restricted plasminogen activators (streptokinases) was in concordance with the host lineage they originally were characterized in. Indications of niche adaptation were also evident in genes mediating immune evasion. Human associated SDSE isolates harbored the immunoglobulin binding Protein G, whereas SDSD appears to rely on either MIG or MAG for this purpose.

To delineate genetic regions potentially mediating host adaptation we performed whole genome comparison of SDSD and SDSE genomes. Due to the limited availability of SDSE isolates of animal origin, we restricted the comparison to human associated SDSE isolates. A total of 17 genetic loci, comprising 40 genes, were found to be unique for and ubiquitously present in SDSD (Table S2). Conversely, 73 genes were specific for human associated SDSE, residing in 19 different genetic regions. The genetic content specific to SDSE displayed high similarity to the strictly human pathogen *S.* *pyogenes*, whereas genes unique to SDSD resembled virulence factors identified predominantly in animal pathogens (Table S2). Seven of these unique loci harbored well recognized virulence factors, including Streptolysin O, C5a-peptidase and the pilus operons (Figure S1). Moreover, these seven genetic loci were in close proximity to previously characterized hotspots for genetic recombination or insertion of mobile genetic elements.

### Mobile genetic elements

Genomes were screened for mobile genetic elements and associated virulence and resistance genes (Table S3). Intact bacteriophages were detected in 81% (63/78) of the SDSD isolates, giving an average of 1.3 bacteriophages per genome (range 0–3). This was a markedly higher prevalence than in human associated SDSE, where 40% (14/35) of the isolates harbored a bacteriophage, average 0.5 per genome (range 0–3) (*p* < 0.0001). This difference between the SD subspecies was also reflected in the carriage of phage-related virulence factors. An average of 1.1 phage-related virulence genes were detected per genome in SDSD versus 0.3 per genome in human SDSE. The streptodornases *mf3* and *sda2* were the most common genes detected in both subspecies, but only SDSD were found to harbor phage-related superantigens, including *speC* (6 isolates), *speK* (7), *speL* (3) and *speM* (3).

Conversely, the prevalence of Integrative Conjugative Elements (ICEs) was significantly higher in SDSE. An average of 2.5 ICEs per genome (range 0–4) were detected in human associated SDSE, compared to 1.6 ICEs in SDSD (range 1–4) (*p* < 0.0001). Carriage of ICE associated resistance genes was generally infrequent but was detected in ten SDSD isolates (2 *tet*(O), 6 *tet*(M) and 4 *lnu*(C), and in six human SDSE isolates (1 *tet*(O), 1 *tet*(M), 1 *mef*(A), and 3 *erm*(A)).

All the SDSD isolates harbored an ICE, Tn5252, equipped with a lactose fermentation operon consisting of 11 genes. Remnants of the ICE and operon was detected in 13 of 35 human SDSE isolates but lacked a conjugation apparatus.

## Discussion

To the best of our knowledge, this is the first comprehensive genomic characterization of *Streptococcus* *dysgalactiae* subspecies *dysgalactiae* (SDSD), and the first study to include isolates of ovine origin. Our findings supported SDSD as a distinct taxonomic entity and revealed several features indicating niche specialization, including the presence of unique virulence factors.

Dissection of the SDSD genomes showed that bovine and ovine isolates formed a tight phylogenetic cluster, displaying a mean value of the ANI of 99% and larger than 98% for all the pairwise comparison. Although the pangenome analysis divided the SDSD isolates largely in accordance with the animal species from which they were isolated, we did not identify any marker genes specific to host. This short evolutionary distance is surprising in light of the markedly different disease phenotypes this pathogen produces in sheep and cattle. Further exploration of the genome sequences identified one gene, *gtfC,* existing in two distinct allelic variants, and their distribution correlated with host of origin. In *S.* *agalactiae*, the *gtfC* gene has been verified to encode a glycosylation enzyme involved in post translational modification of the adhesin Srr, leading to modulation of bacterial adherence to host cells^9^. A similar influence on the adhesive properties in SDSD is plausible, and its potential role in host specificity should be further explored.

SD isolates obtained from pigs, dogs, horses, fish and humans were phylogenetically delineated according to source of isolation. The phylogenetic division thus appears to extend beyond the division into the two subspecies, and points to an adaptive evolution of this bacterial species into several host associated lineages. Previous studies based on seven gene MLST have inferred a similar phylogenetic clustering^4,5^.

Recently, Nishiki et al.^7^ sequenced the first SD isolate from fish, and reported a closer resemblance to SDSD than SDSE. However, their result was influenced by the inclusion *S.* *equi* in the phylogenetic analysis, reducing the basis of the comparison to 126 core genes. Removing *S.* *equi* rendered the phylogenetic landscape concordant with our findings, placing fish isolates within the SDSE group. Koh et al.^10^ also reported that their fish isolate, STREP97-15, clustered with SDSD when using a seven gene multilocus sequencing analysis. Nevertheless, the STREP97-15 isolate is classified as SDSE based on its reported phenotypic characteristics of beta hemolysis and Lancefield group G antigen. This highlights the complexity in delineating the two subspecies of SD, but also underscores that high phylogenetic resolution should be sought when inferring genomic relationships.

Notably, transmission between different host species appeared to be very rare. Supporting this, Acke et al.^11^ did not detect any overlapping MLST-profiles among isolates from cats, dogs and horses, even when these animals had shared the same environment. MLST-types harbored by SDSD-isolates in our study were previously exclusively identified in isolates of bovine origin (MLST-database). However, one isolate obtained from a sheep was identified as SDSE and clustered phylogenetically with the clade of SDSE associated with the porcine host, indicating that the species barrier is not absolute. We have previously published a case of human endocarditis caused by an SDSD-isolate^8^, and in the present phylogenetic analysis, this isolate clustered with isolates of bovine origin. Similarly, a case of a fish handler infected with a presumed piscine SD-isolate has previously been documented in Singapore, and reports of human SD-isolates harboring identical MLST-types as pathogens derived from a pig and a dog has been published in Brazil and Australia, respectively^10,12^. Nevertheless, these case reports appear to represent the exceptions rather than the rule, and zoonotic transmission of this species is likely far less common than previously assumed.

In depth dissection and comparison of the SD genomes presented further indications of niche adaptation, revealing host specific repertoires of virulence factors. SDSD notably lacked the pilus-operons but was equipped with several other tools for adhesion. The fibronectin binding protein FnbA and the fibrinogen binding protein DemA, which were both first described in bovine associated SD-strains, were found to be specific for SDSD in our study^13,14^. In addition, we identified a novel srr/secA2-like operon uniquely present in SDSD strains. Srr has previously been characterized in several streptococcal species, including *S.* *agalactiae*, and is a heavily glycosylated surface protein mediating adhesion to host tissues^9^. The srr-operon encodes its own apparatus for secretion (secA/Y) and post translational glycosylation. The srr-locus in SDSD comprised all the genes necessary for a functional operon, but the role of this locus in SDSD has yet to be investigated.

In the past decades, several studies have investigated the host specific activity of streptococcal virulence factors. McCoy et al. demonstrated that SD-isolates obtained from horses, pigs and humans were only able to activate plasminogen derived from the homologous host^15^. More recently, the plasminogen activator PadA that is functionally limited to activation of bovine and ovine plasminogen was identified in SDSD^16^. In contrast, human-associated SDSE isolates harbor streptokinase, a close homolog of the plasminogen activator in the strictly human pathogen *S.* *pyogenes*^16^. We detected host specific streptokinase-like genes in all our SD isolates, although the homologs in dog and fish associated lineages have not been functionally characterized (Table 1).

Not surprisingly, SD appears to have adapted to encounter different host-specific immune systems. The protein MIG detected in SDSD for instance, binds exclusively bovine immunoglobulins^17^. Conversely, the C5a-peptidase of human-associated SDSE, identical to that of *S.* *pyogenes*, is induced by human serum but not bovine^18^. SDSE isolates of animal origin also appear to harbor host specific genes predicted to have C5a-peptidase and MIG-like activity (data not shown). However, this is based solely on the presence of functional domains, and the properties of these proteins will have to be experimentally verified.

Of particular interest, the majority of the genetic content found to be specific for human associated SDSE displayed high homology to genes harbored by *S.* *pyogenes* (Table S2). Apart from streptokinase and C5a-peptidase, this included the toxins Streptolysin S, Streptolysin O and NAD-glycohydrolase, as well as the pilus operons and several adhesins. SDSD-specific genes, on the other hand, bore closer resemblance to homologs in other animal associated pathogens, such as bovine *S.* *agalactiae* (Table S2). It is interesting that with respect to these pivotal loci, the two SD lineages harbor genes with closer resemblance to fellow host pathogens than each other.

**Figure 7﻿:**
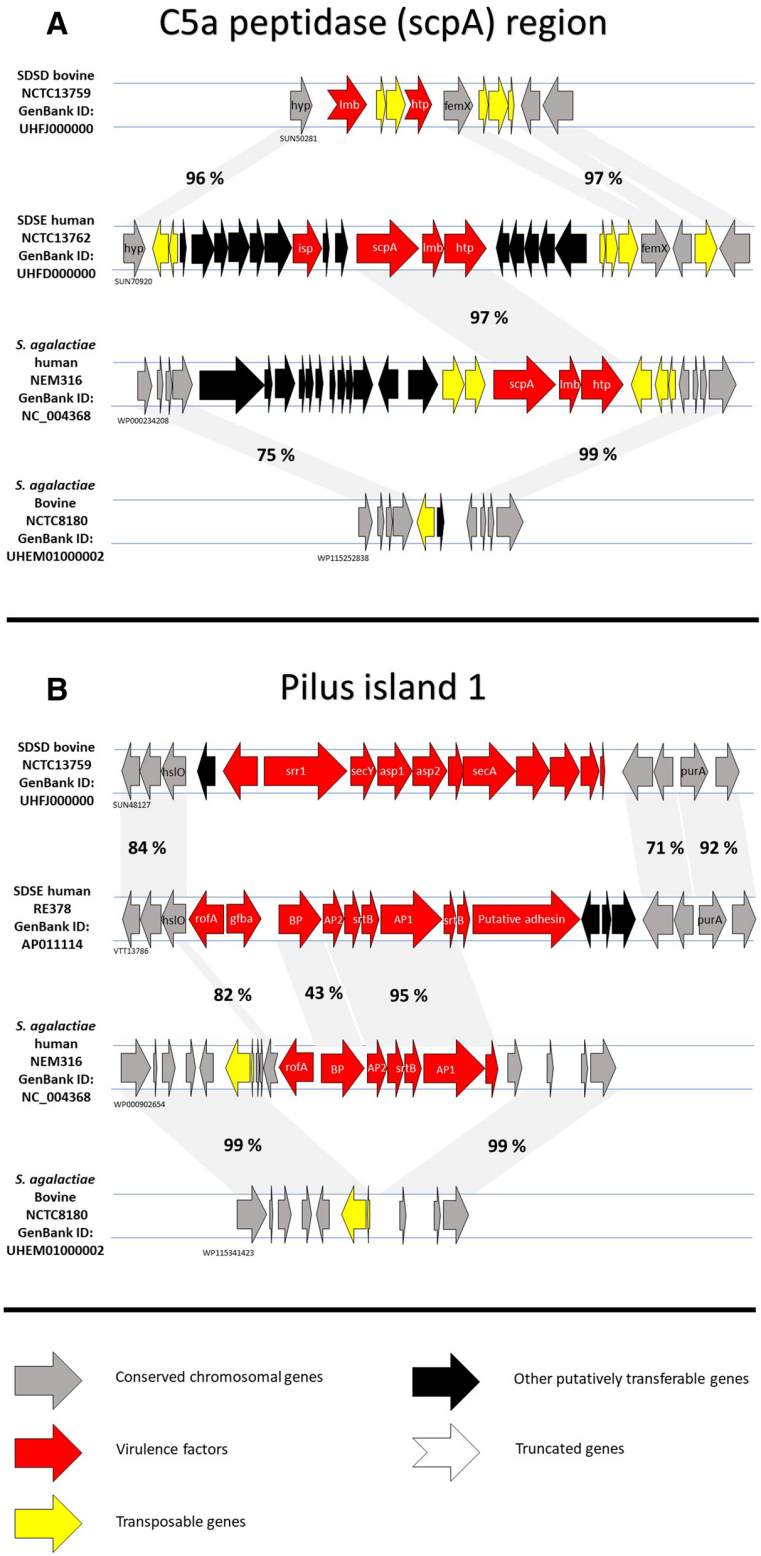
C5a-peptidase and pilus regions in human and bovine SD and *S.* *agalactiae***.** Comparison of genetic features distinguishing bovine and human adapted lineages in *S.* *dysgalactiae* and *S.* *agalactiae*. Panel A depicts the C5a-peptidase region, harboring a highly similar *scpA* and *lmb* gene cassette in human associated SDSE and *S.* *agalactiae*. Pilus regions (Panel B) are absent in bovine SDSD, and bovine *S.* *agalactiae* are also associated with a lack of the pilus 1 operon. Percentages indicate sequence similarity derived from Geneious alignment. The GenBank protein identity for the first gene in each sequence is indicated.

In line with this, several of the genetic features delineating SDSD and human associated SDSE are identical to differences previously noted between bovine and human associated *S.* *agalactiae*, including variable presence of pilus islands and C5a-peptidase (Fig. 7)^19^. Moreover, the acquisition of a novel lactose-fermenting operon (lac2) by bovine mastitis-associated *S.* *agalactiae* was demonstrated to provide a selective growth advantage in a lactose-rich environment such as milk^20^. The lac2-operon was part of a mobile genetic element, and highly similar elements were detected in other streptococcal species, including one bovine associated SDSD isolate^21^. Interestingly, this lactose-operon and its associated mobile genetic element was found to be ubiquitous in our SDSD-isolates, whereas human associated SDSE isolates only harbored a decayed lac2-element. Taken together these findings suggest a similar adaptive pathway in *S.* *agalactiae* and SDSD, but also highlight that interspecies horizontal genetic exchange is likely an important strategy for adaptation to new environments.

In sheep, SDSD preferentially targets the joint tissues rather than the udder, and the potential benefits of harboring a lactose operon are less overt. The mobile genetic element might represent an evolutionary remnant in these pathogens. However, they could potentially benefit from increased capability for cross-species transmission, especially in light of the short phylogenetic distance in general between SDSD isolates of ovine and bovine origin.

Environmental genetic transfer as an adaptive strategy has previously been postulated in *S.* *agalactiae*^22^. SD and *S.* *agalactiae* have overlapping ecological habitats providing ample opportunity for interaction, and conjugative exchange of mobile genetic elements between these two pathogens has been demonstrated in vitro^23^. Notably, we found several of the loci containing lineage-specific genes to be in close proximity to characterized hotspots for insertion of mobile genetic elements or genetic recombination^24^. In one of these hotspots, we uncovered that all the host specific lineages of SD harbored unique genetic contents, including Streptolysin O in the human lineage, DemA in SDSD, streptokinase in pig isolates and different Protein G-like proteins in canine and piscine associated isolates (Figure S2). Taken together, it seems feasible that the host-specific genome in part represents remnants of cargo genes from past encounters with mobile genetic elements, and that bacteriophages and ICE shape the genetic landscape of SD, contributing to the continuous evolution and niche versatility of these pathogens.

We found SDSD to harbor markedly more bacteriophages than SDSE. This was also reflected in the prevalence of phage-mediated virulence factors such as superantigens and mitogenic factors, in line with a previous array study in these pathogens^25^. In fact, except for the chromosomally encoded *speG*, we could not detect superantigens in any of the human associated SDSE genomes. Bacteriophages are abundant in the farm environment, and interspecies transduction within this milieu could facilitate the high phage-infection rate observed in SDSD^26^. However, the biological implications of being equipped with such armory have yet to be elucidated.

Rosinski-Chupin et al.^27^ revealed a reductive evolution to be the most notable in fish-adapted *S.* *agalactiae* variants, primarily comprising deletion and inactivation of several metabolic functions. In piscine SD isolates, disruption of the *emm*-gene operon and pilus island1 by insertion sequences has been reported^7^. Similarly, we observed insertion sequences affecting other virulence factors in SD, including the deletion of the *emm*-gene in most swine associated SD isolates and the Streptolysin S operon in all SDSD isolates (Figure S2). This suggests that these virulence factors are dispensable in certain host environments and agrees with the notion that a combination of gene loss and acquisition are likely to be involved in niche partitioning^28^.

The study is limited by the confined geographic origin of the majority of the SDSD-isolates. However, the phylogenetic clustering and host specific genetic content was conserved also in the genomes procured from public repositories, inferring transferability of our findings to other regions. Nevertheless, future studies involving whole genome sequencing of SDSD-isolates are needed to broaden our understanding of this important pathogen, especially concerning ovine-associated infections. Moreover, characterization of more SD isolates from canine, porcine and piscine sources is warranted to further explore niche specialization and host adaption within this species, and to further refine the taxonomic delineation of SD.

## Conclusion

Using whole genome sequencing we reveal that *Streptococcus* *dysgalactiae* can be delineated into several host specific lineages, and that cross-species transmission appears to be rare. The sublineages are equipped with distinct repertoires of adhesins, toxins and immune evasion proteins likely contributing to host adaption. Moreover, several pivotal genetic loci are in close proximity to hotspots for insertion of mobile genetic elements, suggesting that horizontal genetic transfer could be contributing to niche adaptation and host specificity. The complexity of SD taxonomy is a cause of considerable confusion, and the current subspecies definition could benefit from further scrutiny.

## Materials and methods

### Bacterial genomes included in the study

A total of 156 genomes sequences, 78 SDSD and 78 SDSE, were analyzed in this study (Table S1). Of the 78 SDSD genomes, 60 isolates were sequenced in the present study and were isolated from dairy cows (n = 37) and sheep (n = 23) in Norway as described below. The other genomes were retrieved from public databases or publications (Table S1). Of the SDSE genomes 40 new isolates were sequenced in this study and the remaining genomes were obtained from public databases. The newly sequenced SDSE genomes included 24 isolates from human and 16 isolates from dog (6), horse (4) and pig (6). Genomes sequenced as part of this study are available at DDBJ/ENA/GenBank under the BioProject PRJEB42928 for the SDSD genomes and BioProject PRJEB43000 for SDSE isolates.

### Bovine and ovine SDSD isolates

The bovine and ovine SDSD isolates were collected in a project investigating SDSD diversity in sheep flocks and in bovine dairy herds in Norway (manuscripts in preparation), and the sequence types (ST) of the isolates had already been determined. Ovine isolates were collected between 2016 and 2020 from joint aspirates of lambs with infectious arthritis and body sites of lambs and ewes from 19 sheep flocks. The sheep flocks were located in Northern Norway (n = 14), Western Norway (n = 4) and Eastern Norway (n = 5). One isolate was arbitrarily selected to represent each flock.

Bovine isolates were collected between 2018 and 2020 from quarter milk samples and body sites of cows in eight dairy herds in Eastern Norway. One isolate per ST per herd was arbitrarily selected (range 1–3 STs per herd). In addition, isolates from clinical (n = 10) and subclinical (n = 10) mastitis in dairy cows were randomly selected from the TINE SA mastitis laboratory (Molde, Norway) in the period between March and December 2019. These isolates originated from 20 different dairy herds across the country.

### Culturing conditions and DNA extraction

Bacterial isolates were revived and cultured aerobically overnight on blood agar plates with 5% bovine blood (Oxoid). Genomic DNA was extracted using a MagNA Pure 96 instrument (Roche) and MagNA Pure DNA and NA SV Kit (Roche). One μl of bacterial culture was dissolved in 1 ml of phosphate buffered saline, mixed with Bacterial Lysis Buffer 1:1 and mechanically disrupted, 4 times for 1 min, using FastPrep-24 and 2 ml Lysing Matrix B (MP biomedicals). With an input of 200 µl genomic DNA was extracted using the DNA Blood ds SV protocol optimized for double-stranded DNA and NGS and eluted in 50 µl.

### Genome sequencing and pangenome analysis

Genomic DNA was quantified using the Qubit 3.0 fluorometer (Life Technologies, Waltham, MA USA). DNA was normalized to 0.2 ng/μL and the sequencing library was prepared using the Nextera XT DNA Sample Prep kit (Illumina, San Diego, California, USA) according to the manufacturer´s instructions. Sequencing was performed using the Illumina MiSeq (Illumina, San Diego, California, USA) and V3 chemistry. Raw sequences were quality filtered using Trimmomatic^29^ and de novo assembled using Shovill pipeline (https://github.com/tseemann/shovill). Contigs shorter than 1000 bp and with coverage < 3 were removed prior the annotation step. All the genomes used in this study were annotated using the Prokka pipeline^30^. The protein coding sequences (CDS) were compared with an all-against-all approach, using blastp and the panmatrix was constructed using the R package micropan^31^. CDS were grouped in clusters, using a similarity threshold of 0.75 and complete linkage using the function “bClust” from the micropan package^31^. The R package “micropan” was used to compute openness and closeness of the genomes using Heaps´ law implemented in the function “heaps”. The alpha parameter was calculated for all the genomes included in the analysis and for genomes belonging to the two different subspecies of SD*.* Distances between genomes was calculated from the presence/absence panmatrix by clustering the genomes using Manhattan distances and visualized using the R packages Dendextend^32^.

### Multilocus sequence typing and phylogenetic analysis

Typing of the isolates was performed using the MLST 2.0 software available at the Center for Genomic Epidemiology webpage (http://www.genomicepidemiology.org/)^33^, and novel sequence-types were submitted to the MLST-database (pubmlst.org). The phylogenetic relationship between all SD isolates from the current study (Table S1) was determined using single orthologous genes (defined as genes present in only one copy per genome and obtained from the pangenome analysis). For all gene clusters containing single orthologous genes, present in all genomes, the nucleotide sequences were translated to amino acids, aligned using “Decipher” r-package^34^, and back-translated to nucleotide sequences. All alignments were then concatenated into a single file containing all the aligned, single-copy, orthologous genes. Positions with gaps and indels were removed from the final fasta file. A Maximum likelihood tree was constructed using the Geneious software V 10.0.7. with Jukes–Cantor distance, four substitution rate categories and empirically determined gamma substitution parameter with a bootstrap of 100. A second method based on average nucleotide identity (ANI) was performed to compare the genomes using the fastANI algorithm^35^. Clustering of the pairwise comparison of ANI results was constructed using Euclidian distances.

### Characterization of virulome, resistome and mobilome

All the genomes were screened for streptococcal virulence factors and resistance genes using Geneious. Bacteriophages were detected using Phaster^36^, and Integrative Conjugative Elements (ICE) were identified by a combination of BLAST search and manual inspection of integration hotspots, as previously described^24^. Mann–Whitney U test was used to compare the distribution and quantity of mobile genetic elements in SDSD and SDSE.

In order to locate regions potentially involved in host adaptation, genomes of isolates derived from different host were manually compared and inspected for unique genomic content. The contigs of each individual genome were first sorted by alignment to a reference genome using the MAUVE MCM algorithm^37^. NCTC13759 and NCTC13762 were used as reference for SDSD and SDSE, respectively. The sorted contigs were concatenated, and whole genomes were aligned for comparison using the progressive MAUVE-algorithm. Putatively unique genes and genetic regions were verified through BLAST search against all the genomes. Novel and hypothetical genes were checked for conserved functional domains using the NCBI Conserved Domain BLAST service^38^.

### Ethical approval

Human isolates were obtained from a study which underwent institutional ethics review and approval (2019/63132 Regional Ethics Committee West, Norway). Farms included in this study operate under the regulations of the Norwegian Food Safety Authority regarding food production and animal care. The farmers provided permission for the sampling and for the use of their information in this study. All methods were carried out in accordance with relevant guidelines and regulations. Invasive samples (joint aspirates) were only collected from sick animals and the sampling was performed by veterinarians in clinical practice as part of the routine diagnostic work, which does not require ethical approval.

## Supplementary Information

Supplementary Information 1.

Supplementary Information 2.

Supplementary Information 3.

Supplementary Information 4.

Supplementary Information 5.

Supplementary Information 6.

## References

[1] Vieira VV (1998). Genetic relationships among the different phenotypes of *Streptococcus**dysgalactiae* strains. Int. J. Syst. Bacteriol..

[2] Smistad M (2020). Flock-level risk factors for outbreaks of infectious arthritis in lambs, Norway 2018. Acta Vet. Scand..

[3] Østerås, O. HELSEKORTORDNINGEN, STORFE 2018—STATISTIKKSAMLING https://medlem.tine.no/fagprat/husdyrkontrollen/_attachment/499149?_ts=170afd0cd3d (2020).

[4] Jensen A, Kilian M (2012). Delineation of *Streptococcus**dysgalactiae*, its subspecies, and its clinical and phylogenetic relationship to *Streptococcus**pyogenes*. J. Clin. Microbiol..

[5] Pinho MD (2016). Beta-hemolytic *Streptococcus**dysgalactiae* strains isolated from horses are a genetically distinct population within the *Streptococcus**dysgalactiae* taxon. Sci. Rep. UK.

[6] Velez JR (2017). Whole-genome sequence analysis of antimicrobial resistance genes in *Streptococcus**uberis* and *Streptococcus**dysgalactiae* isolates from Canadian dairy herds. Front. Vet. Sci..

[7] Nishiki I, Yoshida T, Fujiwara A (2019). Complete genome sequence and characterization of virulence genes in Lancefield group C *Streptococcus**dysgalactiae* isolated from farmed amberjack (Seriola dumerili). Microbiol. Immunol..

[8] Jordal S, Glambek M, Oppegaard O, Kittang BR (2015). New tricks from an old cow: Infective endocarditis caused by *Streptococcus**dysgalactiae* subsp *dysgalactiae*. J. Clin. Microb..

[9] Mistou MY, Dramsi S, Brega S, Poyart C, Trieu-Cuot P (2009). Molecular dissection of the secA2 locus of group B *Streptococcus* reveals that glycosylation of the *Srr1* LPXTG protein is required for full virulence. J. Bacteriol..

[10] Koh TH, Rahman NBA, Sessions OM (2020). Comparative genomic analysis of *Streptococcus**dysgalactiae* subspecies *dysgalactiae*, an occasional cause of zoonotic infection. Pathology.

[11] Acke E (2015). Prevalence of *Streptococcus**dysgalactiae* subsp. equisimilis and *S.**equi* subsp zooepidemicus in a sample of healthy dogs, cats and horses. N. Z. Vet. J..

[12] Schrieber L, Towers R, Muscatello G, Speare R (2014). Transmission of *Streptococcus**dysgalactiae* subsp *equisimilis* between child and dog in an aboriginal Australian community. Zoonoses Public Health.

[13] Lindgren PE (1993). Two different genes coding for fibronectin-binding proteins from *Streptococcus**dysgalactiae*. The complete nucleotide sequences and characterization of the binding domains. Eur. J. Biochem..

[14] Vasi J, Frykberg L, Carlsson LE, Lindberg M, Guss B (2000). M-like proteins of *Streptococcus**dysgalactiae*. Infect. Immun..

[15] McCoy HE, Broder CC, Lottenberg R (1991). Streptokinases produced by pathogenic group C streptococci demonstrate species-specific plasminogen activation. J. Infect. Dis..

[16] Ward PN, Abu-Median ABAK, Leigh JA (2008). Structural consideration of the formation of the activation complex between the staphylokinase-like streptococcal plasminogen activator PadA and bovine plasminogen. J. Mol. Biol..

[17] Song XM, Perez-Casal J, Fontaine MC, Potter AA (2002). Bovine immunoglobulin A (IgA)-binding activities of the surface-expressed Mig protein of *Streptococcus**dysgalactiae*. Microbiology (Reading).

[18] Gleich-Theurer U (2009). Human serum induces streptococcal c5a peptidase expression. Infect. Immun..

[19] Sorensen UB, Poulsen K, Ghezzo C, Margarit I, Kilian M (2010). Emergence and global dissemination of host-specific *Streptococcus**agalactiae* clones. MBio.

[20] Richards VP, Choi SC, Pavinski Bitar PD, Gurjar AA, Stanhope MJ (2013). Transcriptomic and genomic evidence for *Streptococcus**agalactiae* adaptation to the bovine environment. BMC Genomics.

[21] Richards VP (2011). Comparative genomics and the role of lateral gene transfer in the evolution of bovine adapted *Streptococcus**agalactiae*. Infect. Genet. Evol..

[22] Richards VP (2019). Population gene introgression and high genome plasticity for the zoonotic pathogen *Streptococcus**agalactiae*. Mol. Biol. Evol..

[23] Haenni M (2010). Diversity and mobility of integrative and conjugative elements in bovine isolates of *Streptococcus**agalactiae*, *S.**dysgalactiae* subsp *dysgalactiae*, and *S.**uberis*. Appl. Environ. Microbiol..

[24] Ambroset C (2016). New insights into the classification and integration specificity of *Streptococcus* integrative conjugative elements through extensive genome exploration. Front. Microbiol..

[25] Rato MG (2011). Virulence gene pool detected in bovine group C *Streptococcus**dysgalactiae* subsp *dysgalactiae* isolates by use of a group A *S.**pyogenes* virulence microarray. J. Clin. Microbiol..

[26] Marco MB, Moineau S, Quiberoni A (2012). Bacteriophages and dairy fermentations. Bacteriophage.

[27] Rosinski-Chupin I (2013). Reductive evolution in *Streptococcus**agalactiae* and the emergence of a host adapted lineage. BMC Genomics.

[28] Almeida A (2016). Persistence of a dominant bovine lineage of group B *Streptococcus* reveals genomic signatures of host adaptation. Environ. Microbiol..

[29] Bolger AM, Lohse M, Usadel B (2014). Trimmomatic: A flexible trimmer for Illumina sequence data. Bioinformatics.

[30] Seemann T (2014). Prokka: Rapid prokaryotic genome annotation. Bioinformatics.

[31] Snipen L, Liland KH (2015). micropan: An R-package for microbial pan-genomics. BMC Bioinform..

[32] Galili T (2015). dendextend: An R package for visualizing, adjusting and comparing trees of hierarchical clustering. Bioinformatics.

[33] Larsen MV (2012). Multilocus sequence typing of total-genome-sequenced bacteria. J. Clin. Microbiol..

[34] Wright ES (2016). Using DECIPHER v2.0 to analyze big biological sequence data in R. R J..

[35] Jain C, Rodriguez-R LM, Phillippy AM, Konstantinidis KT, Aluru S (2018). High throughput ANI analysis of 90K prokaryotic genomes reveals clear species boundaries. Nat. Commun..

[36] Arndt D (2016). PHASTER: A better, faster version of the PHAST phage search tool. Nucleic Acids Res..

[37] Darling AE, Mau B, Perna NT (2010). progressiveMauve: Multiple Geno@me alignment with gene gain, loss and rearrangement. PLoS ONE.

[38] Marchler-Bauer A (2017). CDD/SPARCLE: Functional classification of proteins via subfamily domain architectures. Nucleic Acids Res..

