# Supplementary material for: Whole genome sequencing reveals possible host species adaptation of Streptococcusdysgalactiae
Source: Sci Rep. 2021 Aug 30;11:17350. doi: 10.1038/s41598-021-96710-z (PMC8405622; doi:10.1038/s41598-021-96710-z)
Supplement: Supplementary file 1 — Supplementary Information 1. [file 41598_2021_96710_MOESM1_ESM.pptx]

## Slide 1
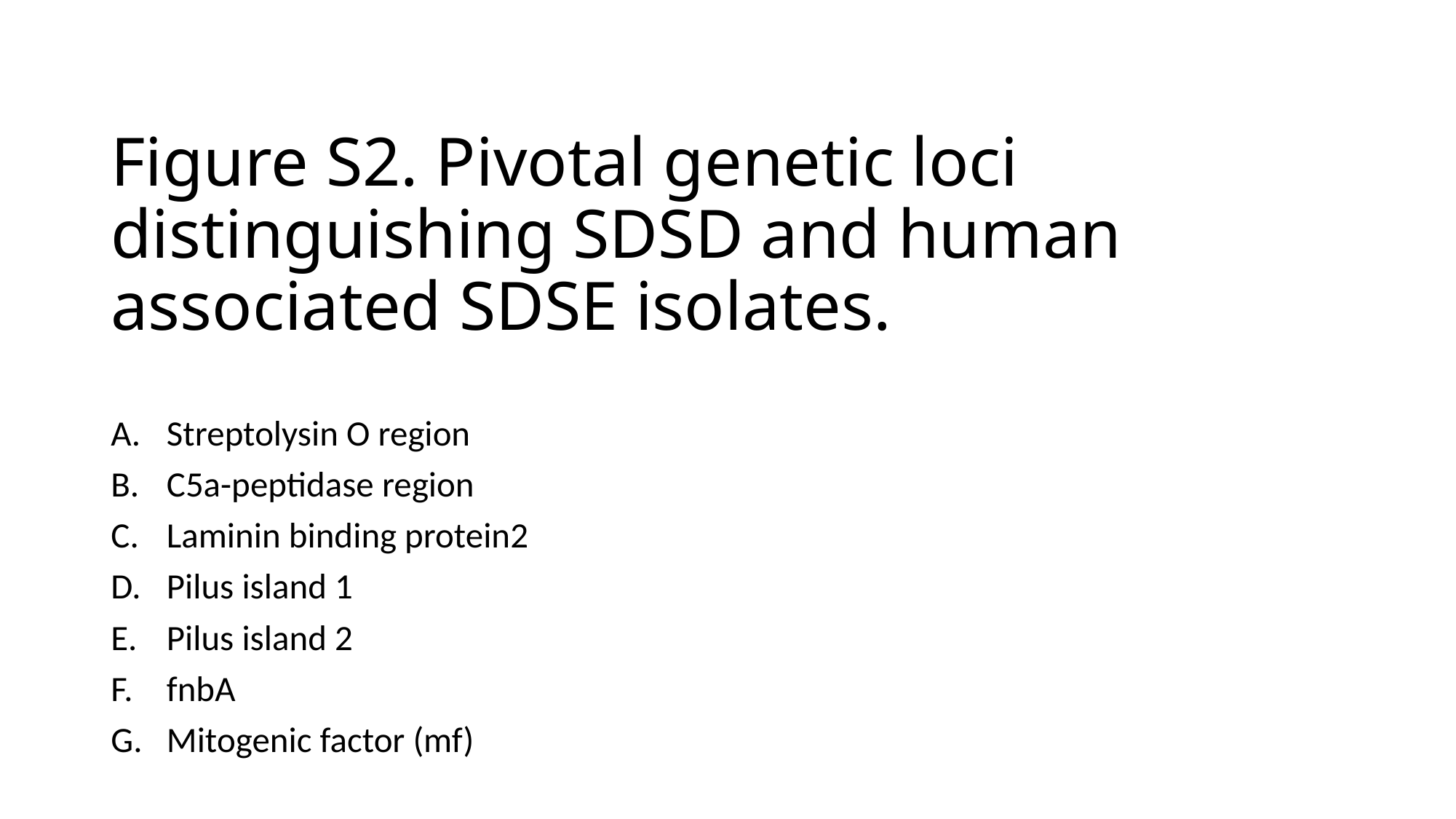

# Figure S2. Pivotal genetic loci distinguishing SDSD and human associated SDSE isolates.
Streptolysin O region
C5a-peptidase region
Laminin binding protein2
Pilus island 1
Pilus island 2
fnbA
Mitogenic factor (mf)

## Slide 2
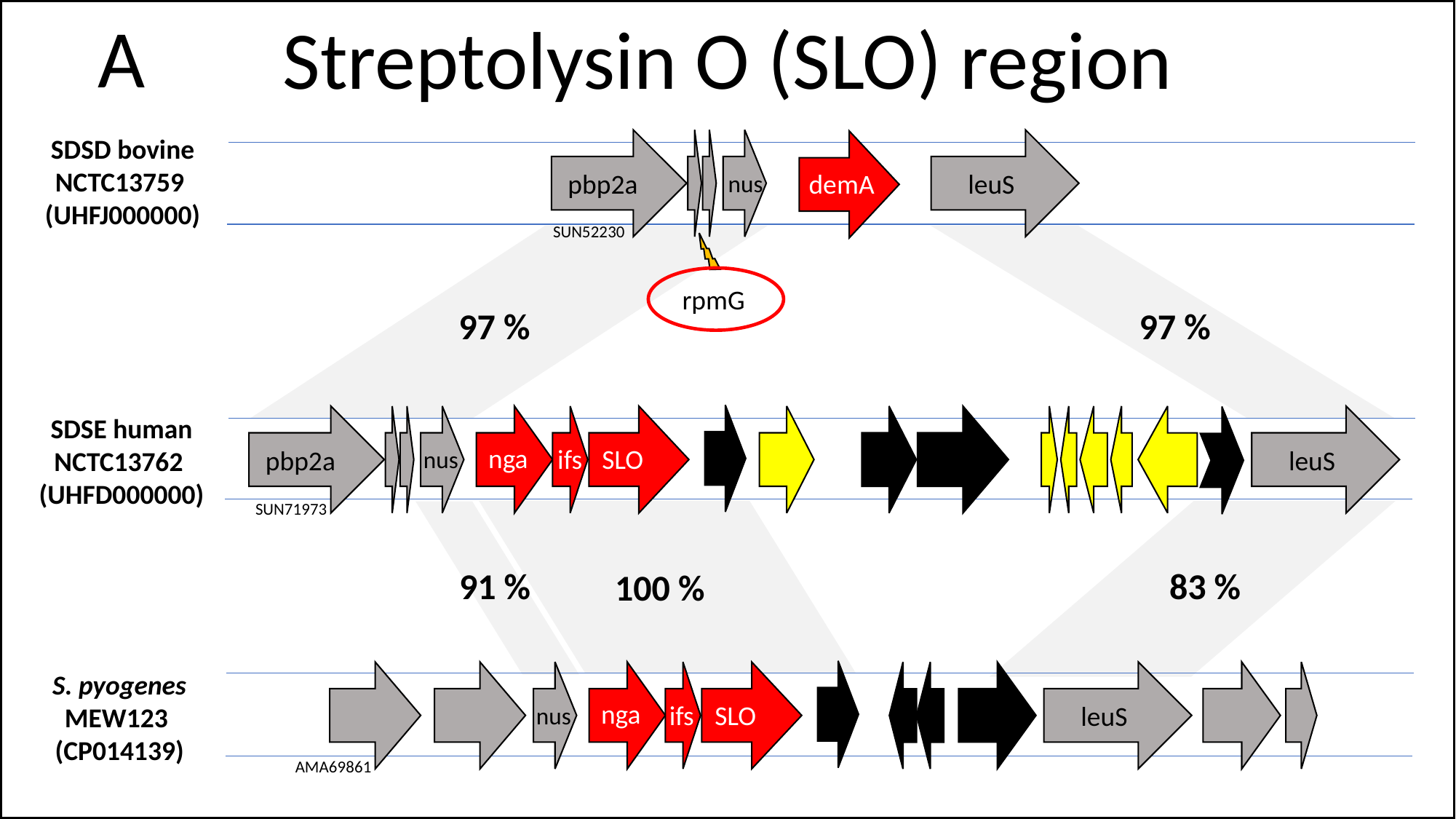

A
Streptolysin O (SLO) region
SDSD bovine
NCTC13759
(UHFJ000000)
pbp2a
demA
leuS
nus
SUN52230
rpmG
97 %
97 %
SDSE human
NCTC13762
(UHFD000000)
nga
ifs
SLO
pbp2a
leuS
nus
SUN71973
91 %
83 %
100 %
S. pyogenes
MEW123
(CP014139)
nga
ifs
SLO
leuS
nus
AMA69861

## Slide 3
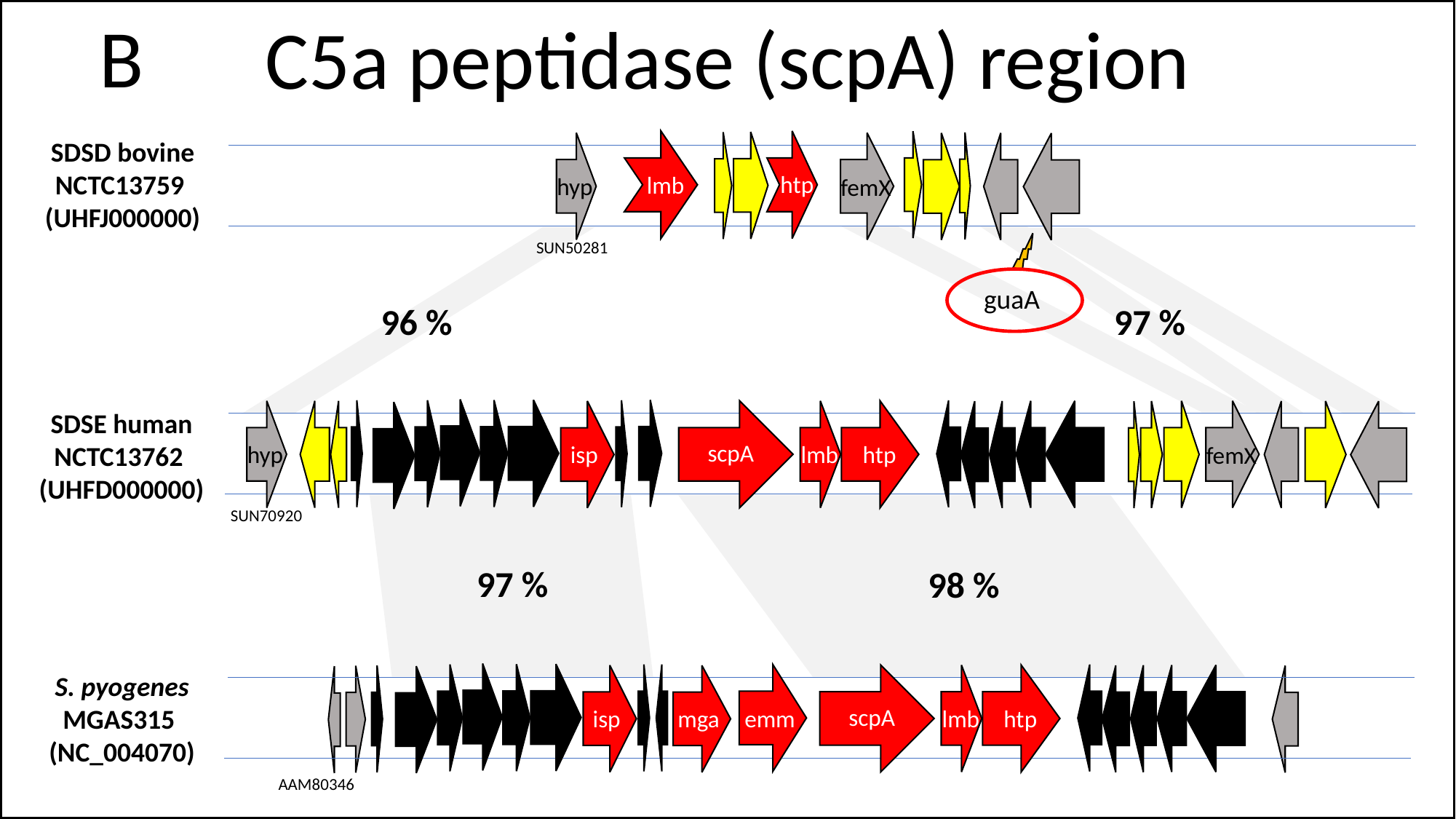

B
C5a peptidase (scpA) region
SDSD bovine
NCTC13759
(UHFJ000000)
htp
lmb
hyp
femX
SUN50281
guaA
97 %
96 %
SDSE human
NCTC13762
(UHFD000000)
scpA
lmb
htp
hyp
isp
femX
SUN70920
97 %
98 %
S. pyogenes
MGAS315
(NC_004070)
scpA
lmb
htp
isp
mga
emm
AAM80346

## Slide 4
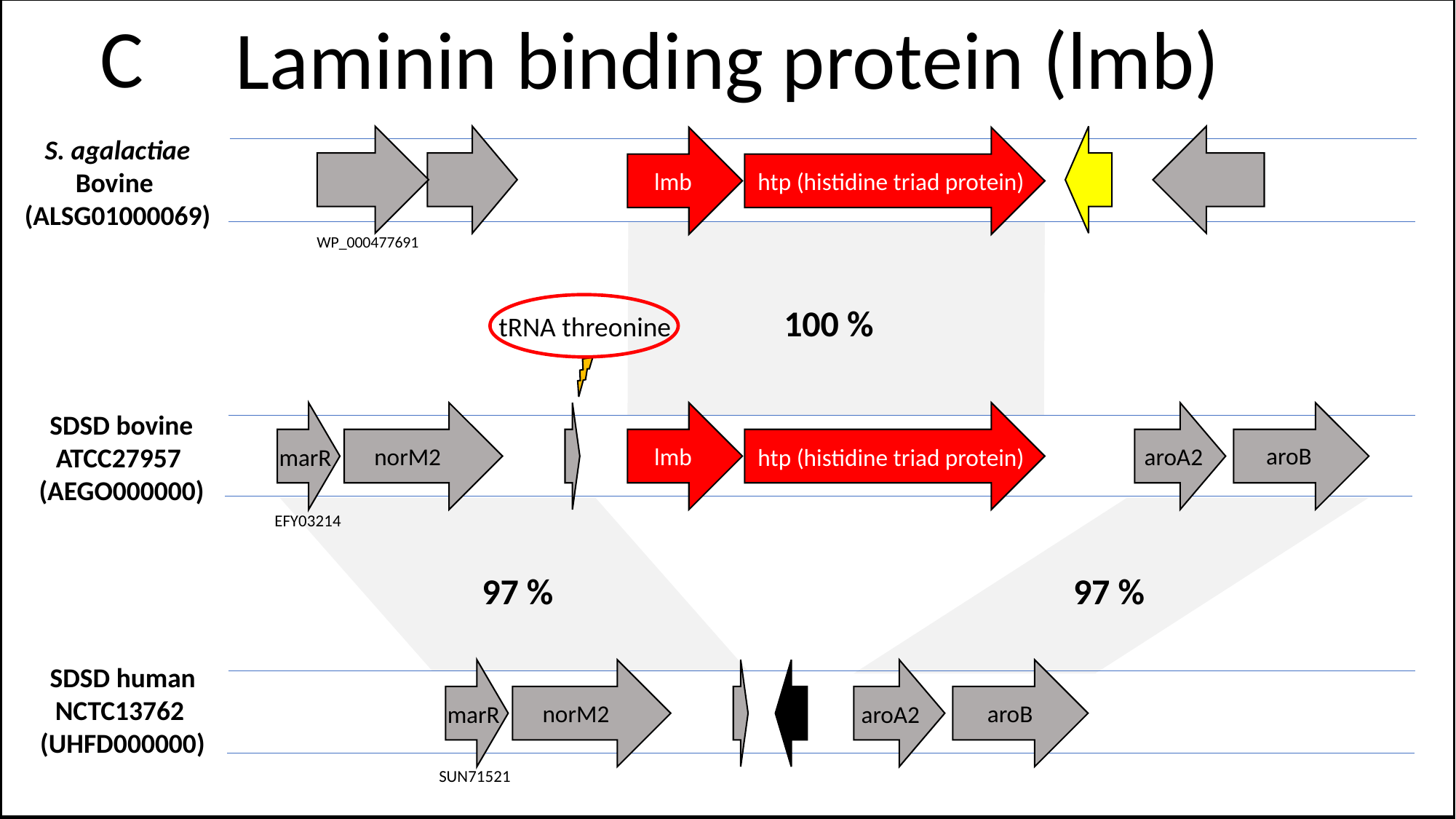

C
Laminin binding protein (lmb)
S. agalactiae
Bovine
(ALSG01000069)
lmb
htp (histidine triad protein)
WP_000477691
100 %
tRNA threonine
SDSD bovine
ATCC27957
(AEGO000000)
aroB
norM2
lmb
aroA2
marR
htp (histidine triad protein)
EFY03214
97 %
97 %
SDSD human
NCTC13762
(UHFD000000)
norM2
aroB
marR
aroA2
SUN71521

## Slide 5
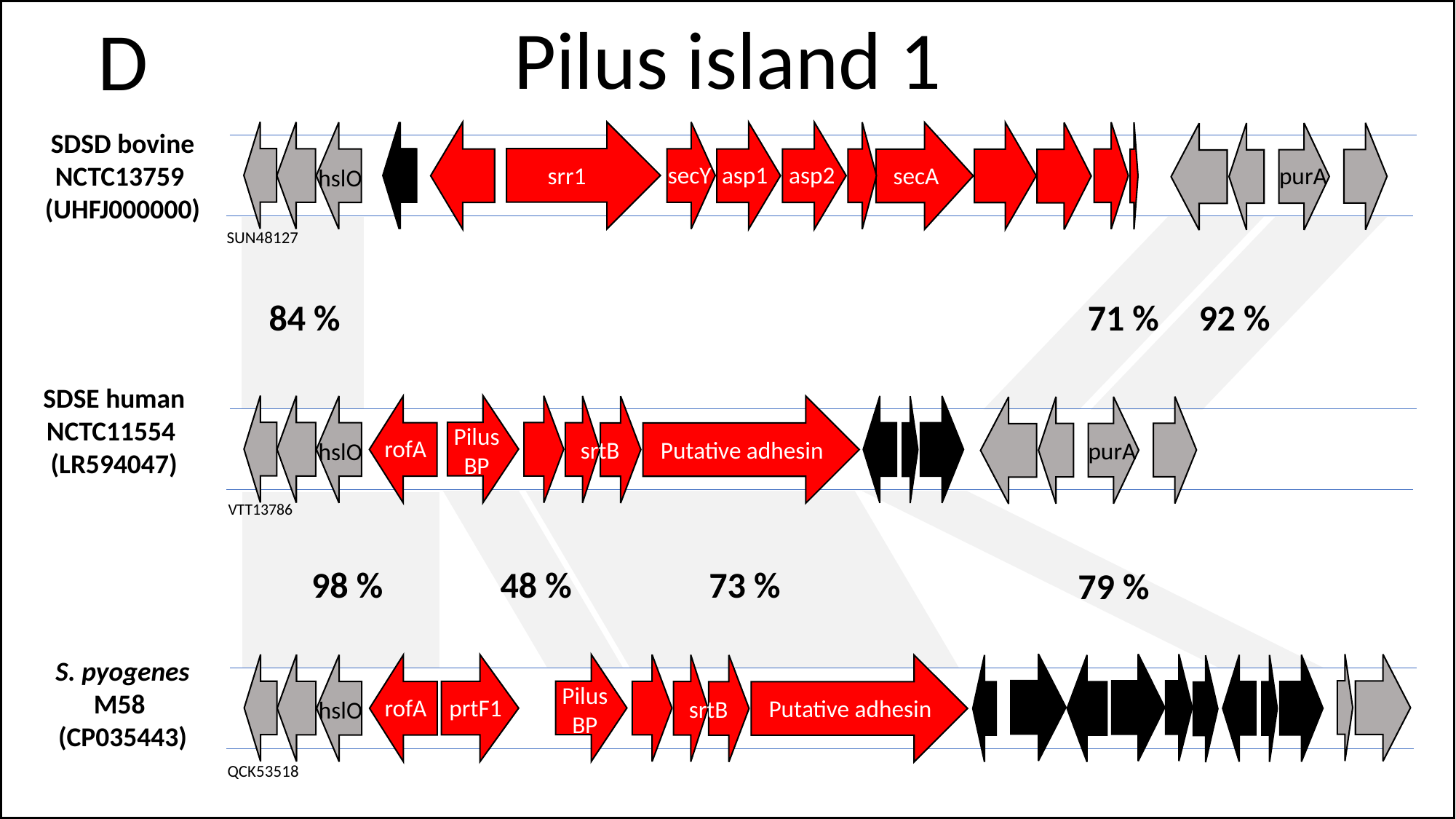

Pilus island 1
D
SDSD bovine
NCTC13759
(UHFJ000000)
asp1
secY
asp2
purA
srr1
secA
hslO
SUN48127
84 %
92 %
71 %
SDSE human
NCTC11554
(LR594047)
Pilus BP
rofA
Putative adhesin
srtB
purA
hslO
VTT13786
98 %
73 %
48 %
79 %
S. pyogenes
M58
(CP035443)
Pilus BP
rofA
prtF1
Putative adhesin
srtB
hslO
QCK53518

## Slide 6
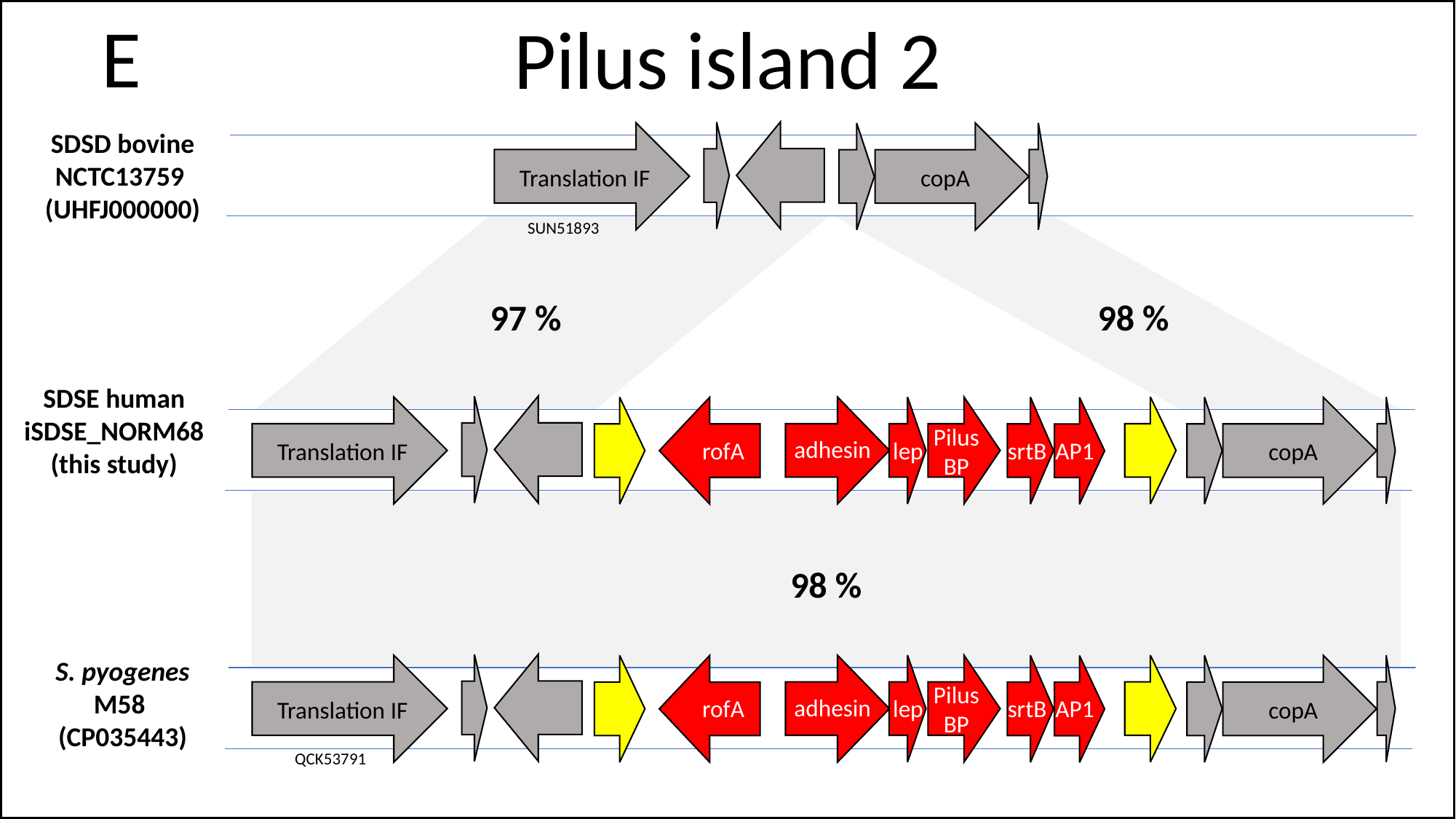

E
Pilus island 2
SDSD bovine
NCTC13759
(UHFJ000000)
Translation IF
copA
SUN51893
97 %
98 %
SDSE human
iSDSE_NORM68
(this study)
Pilus BP
adhesin
rofA
lep
srtB
AP1
Translation IF
copA
98 %
S. pyogenes
M58
(CP035443)
Pilus BP
adhesin
rofA
lep
srtB
AP1
Translation IF
copA
 QCK53791

## Slide 7
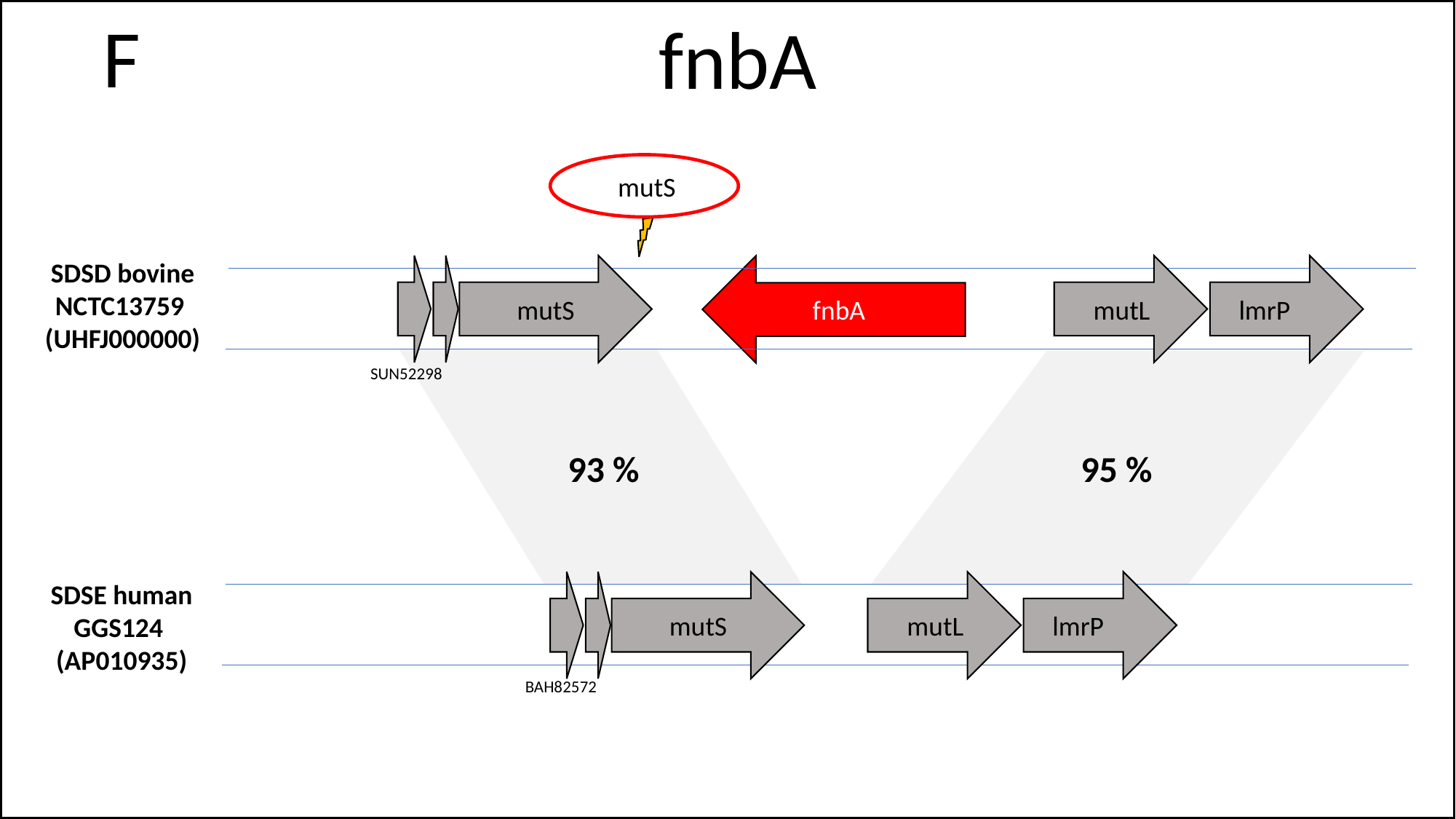

F
fnbA
mutS
SDSD bovine
NCTC13759
(UHFJ000000)
lmrP
mutS
fnbA
mutL
SUN52298
93 %
95 %
SDSE human
GGS124
(AP010935)
lmrP
mutS
mutL
 BAH82572

## Slide 8
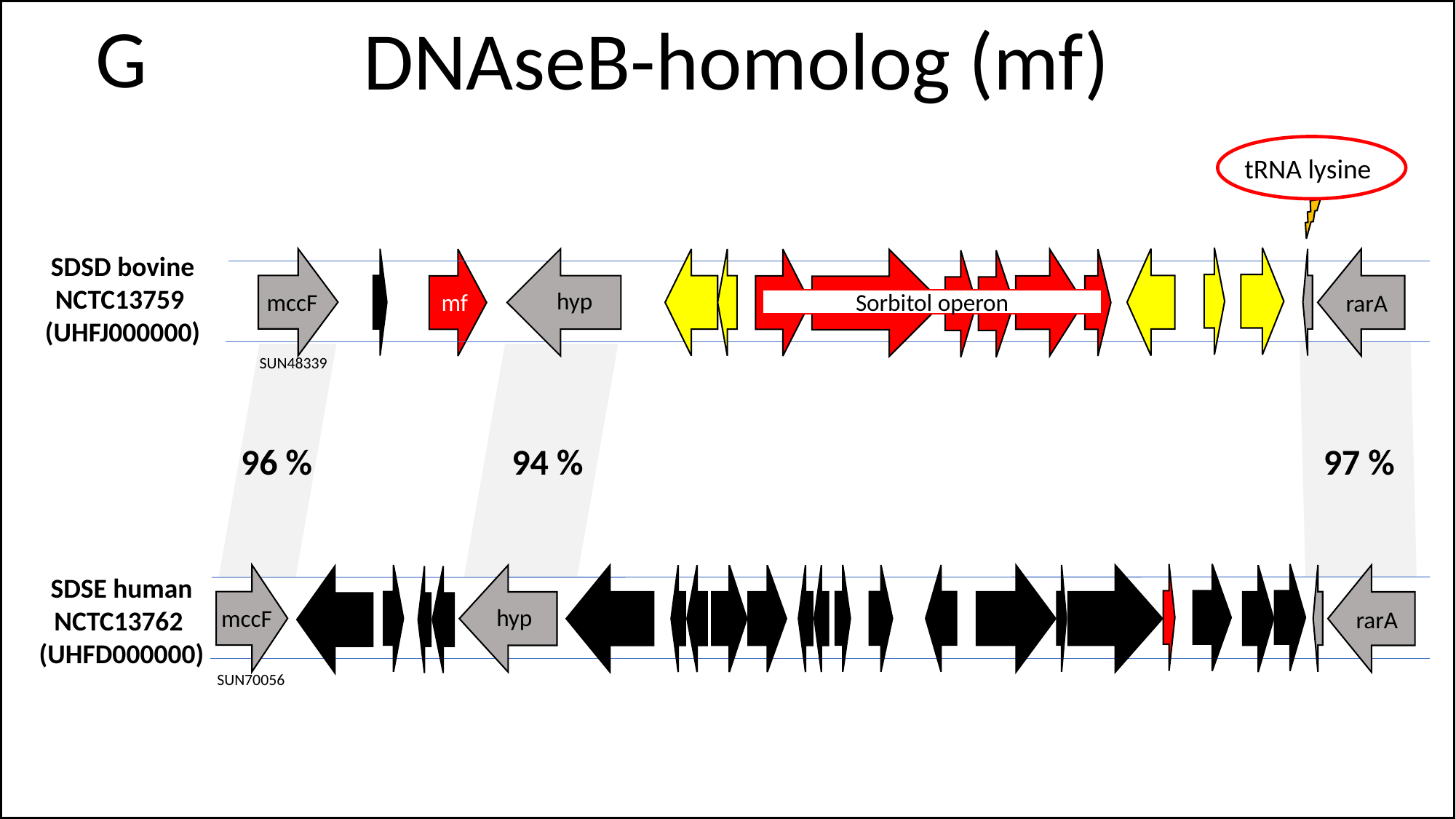

G
DNAseB-homolog (mf)
tRNA lysine
SDSD bovine
NCTC13759
(UHFJ000000)
hyp
mccF
mf
Sorbitol operon
rarA
SUN48339
96 %
94 %
97 %
SDSE human
NCTC13762
(UHFD000000)
hyp
mccF
rarA
SUN70056

## Slide 9
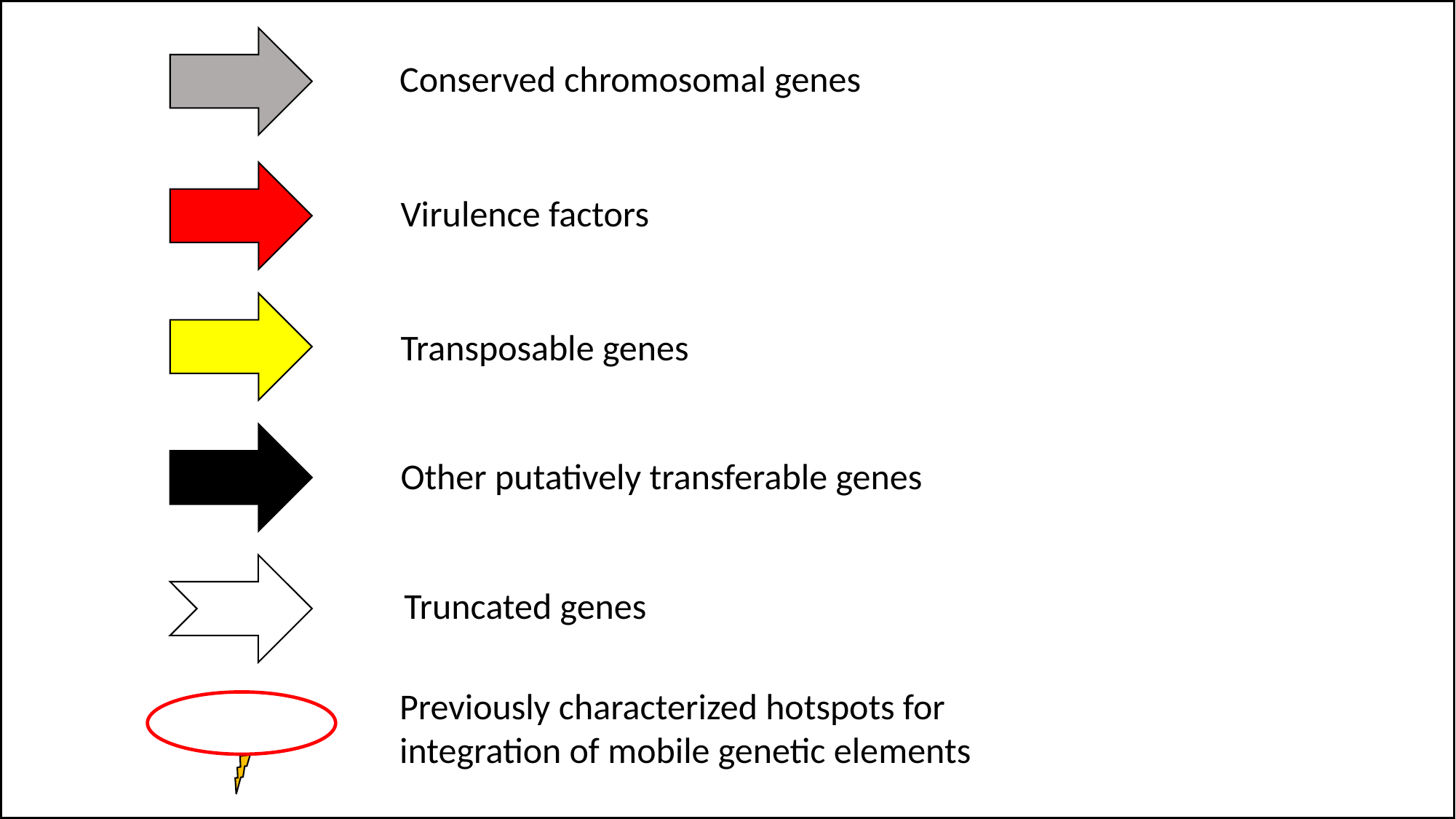

Conserved chromosomal genes
Virulence factors
Transposable genes
Other putatively transferable genes
Truncated genes
Previously characterized hotspots for integration of mobile genetic elements
