# Supplementary material for: Whole genome sequencing reveals possible host species adaptation of Streptococcusdysgalactiae
Source: Sci Rep. 2021 Aug 30;11:17350. doi: 10.1038/s41598-021-96710-z (PMC8405622; doi:10.1038/s41598-021-96710-z)
Supplement: Supplementary file 2 — Supplementary Information 2. [file 41598_2021_96710_MOESM2_ESM.pptx]

## Slide 1
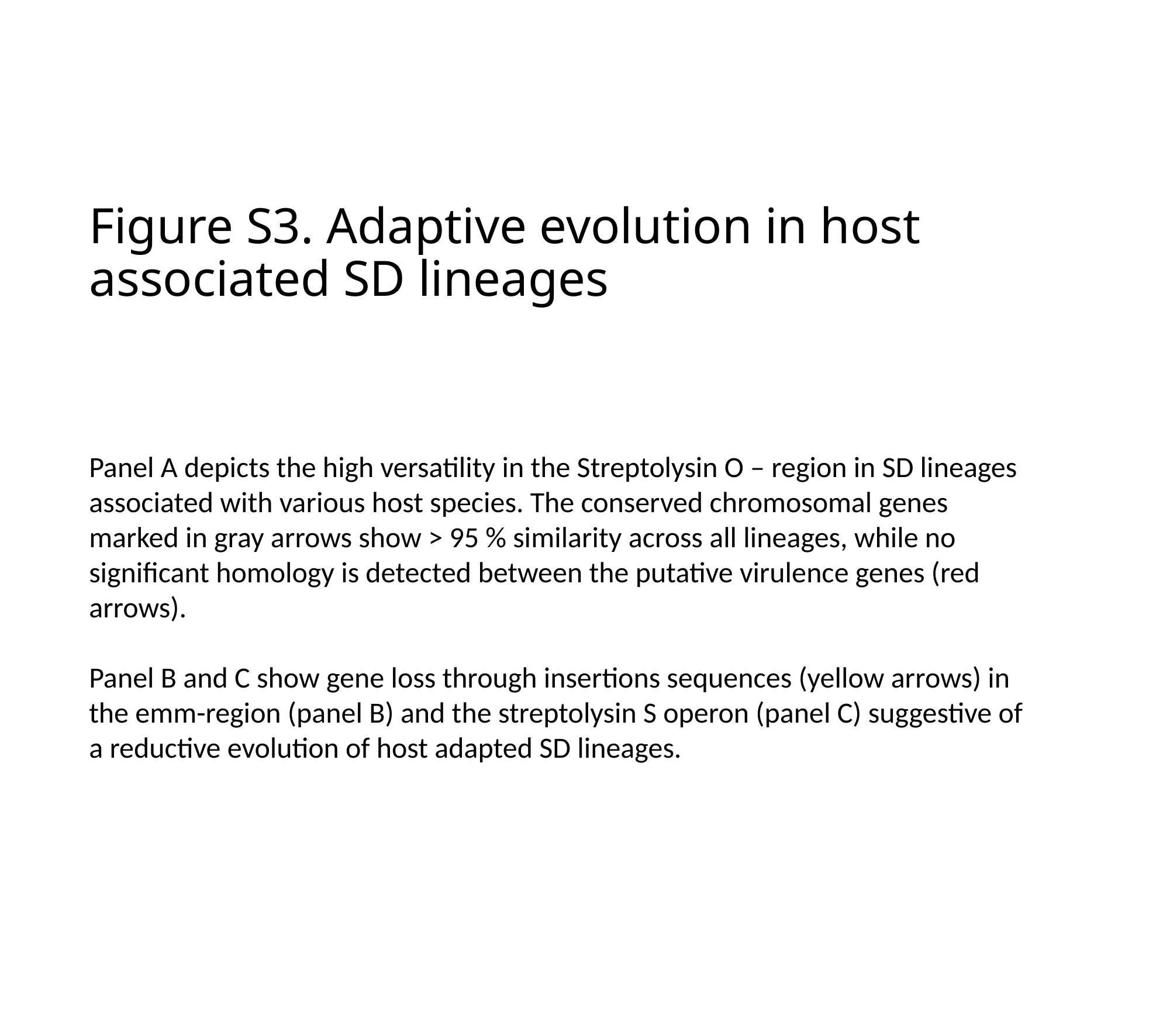

# Figure S3. Adaptive evolution in host associated SD lineages
Panel A depicts the high versatility in the Streptolysin O – region in SD lineages associated with various host species. The conserved chromosomal genes marked in gray arrows show > 95 % similarity across all lineages, while no significant homology is detected between the putative virulence genes (red arrows).
Panel B and C show gene loss through insertions sequences (yellow arrows) in the emm-region (panel B) and the streptolysin S operon (panel C) suggestive of a reductive evolution of host adapted SD lineages.

## Slide 2
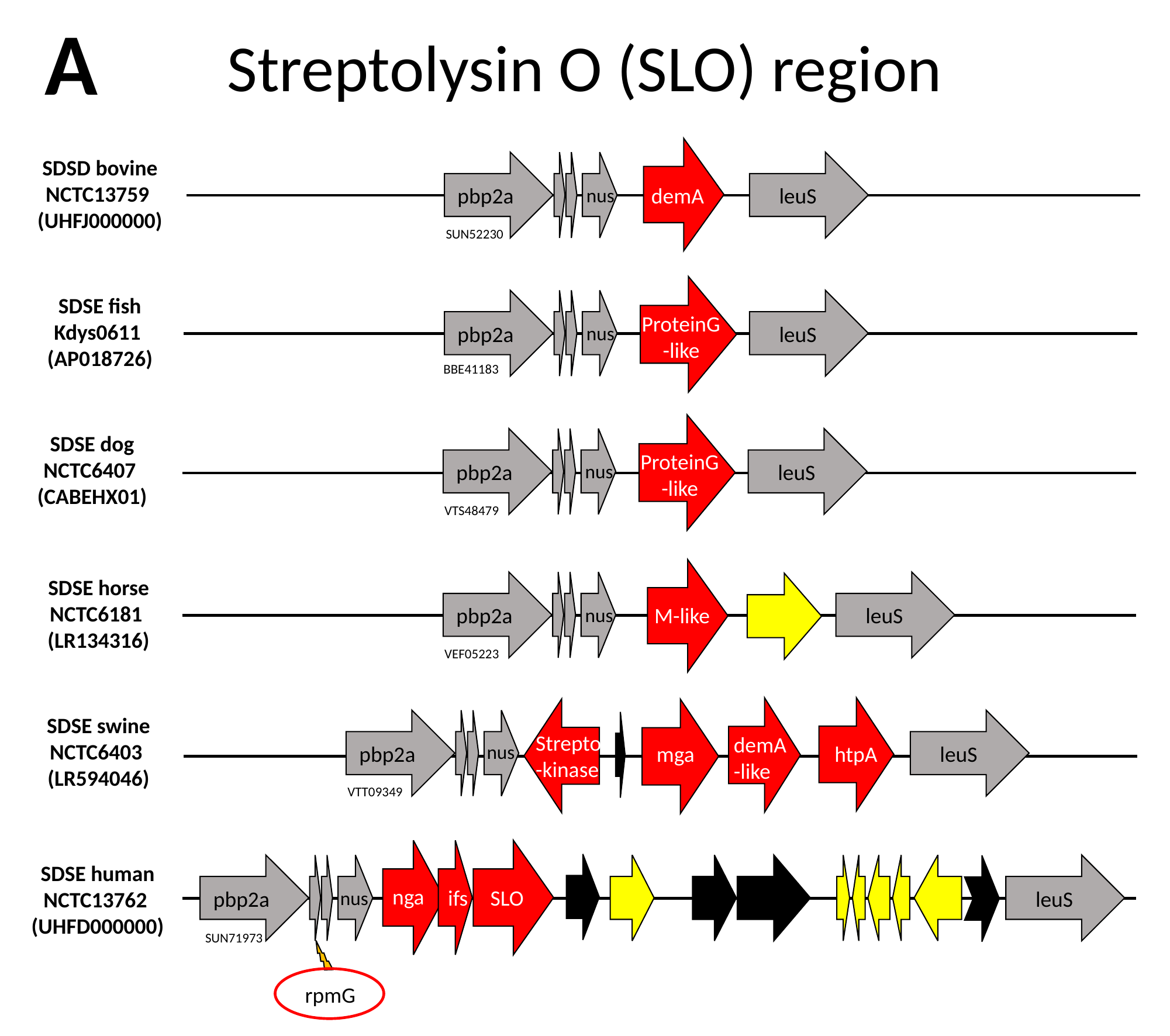

A
Streptolysin O (SLO) region
SDSD bovine
NCTC13759
(UHFJ000000)
pbp2a
demA
leuS
nus
SUN52230
SDSE fish
Kdys0611
(AP018726)
ProteinG
-like
pbp2a
leuS
nus
BBE41183
SDSE dog
NCTC6407
(CABEHX01)
ProteinG
-like
pbp2a
leuS
nus
VTS48479
SDSE horse
NCTC6181
(LR134316)
M-like
pbp2a
leuS
nus
VEF05223
SDSE swine
NCTC6403
(LR594046)
Strepto
-kinase
demA-like
htpA
mga
pbp2a
nus
leuS
VTT09349
SDSE human
NCTC13762
(UHFD000000)
nga
ifs
SLO
pbp2a
leuS
nus
SUN71973
rpmG

## Slide 3
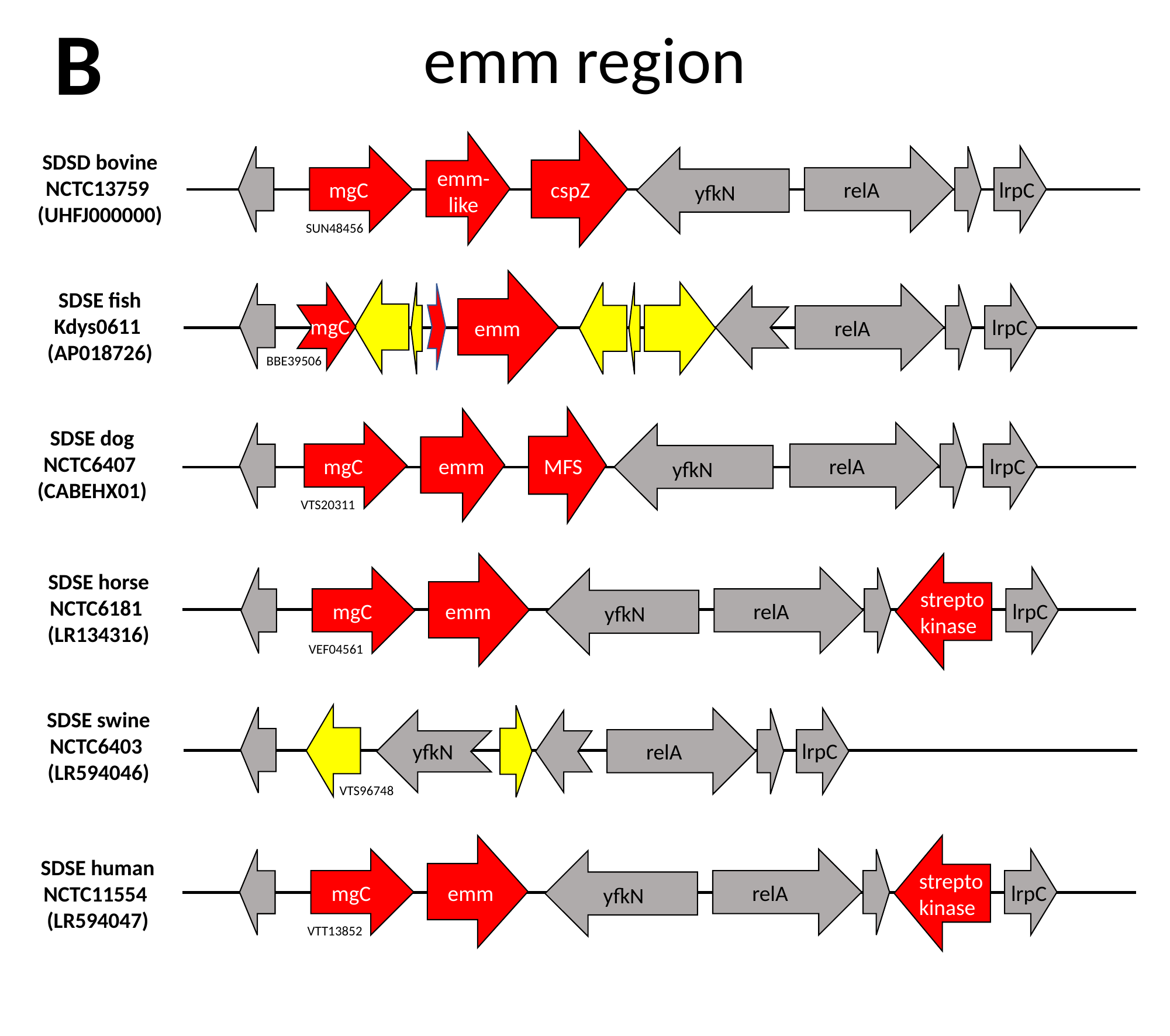

B
emm region
SDSD bovine
NCTC13759
(UHFJ000000)
emm-like
mgC
relA
lrpC
cspZ
yfkN
SUN48456
SDSE fish
Kdys0611
(AP018726)
ProteinG
-like
mgC
lrpC
emm
relA
BBE39506
SDSE dog
NCTC6407
(CABEHX01)
emm
mgC
relA
lrpC
MFS
yfkN
VTS20311
SDSE horse
NCTC6181
(LR134316)
streptokinase
emm
relA
lrpC
mgC
yfkN
VEF04561
SDSE swine
NCTC6403
(LR594046)
ProteinG
-like
lrpC
yfkN
relA
VTS96748
SDSE human
NCTC11554
(LR594047)
streptokinase
emm
relA
lrpC
mgC
yfkN
VTT13852

## Slide 4
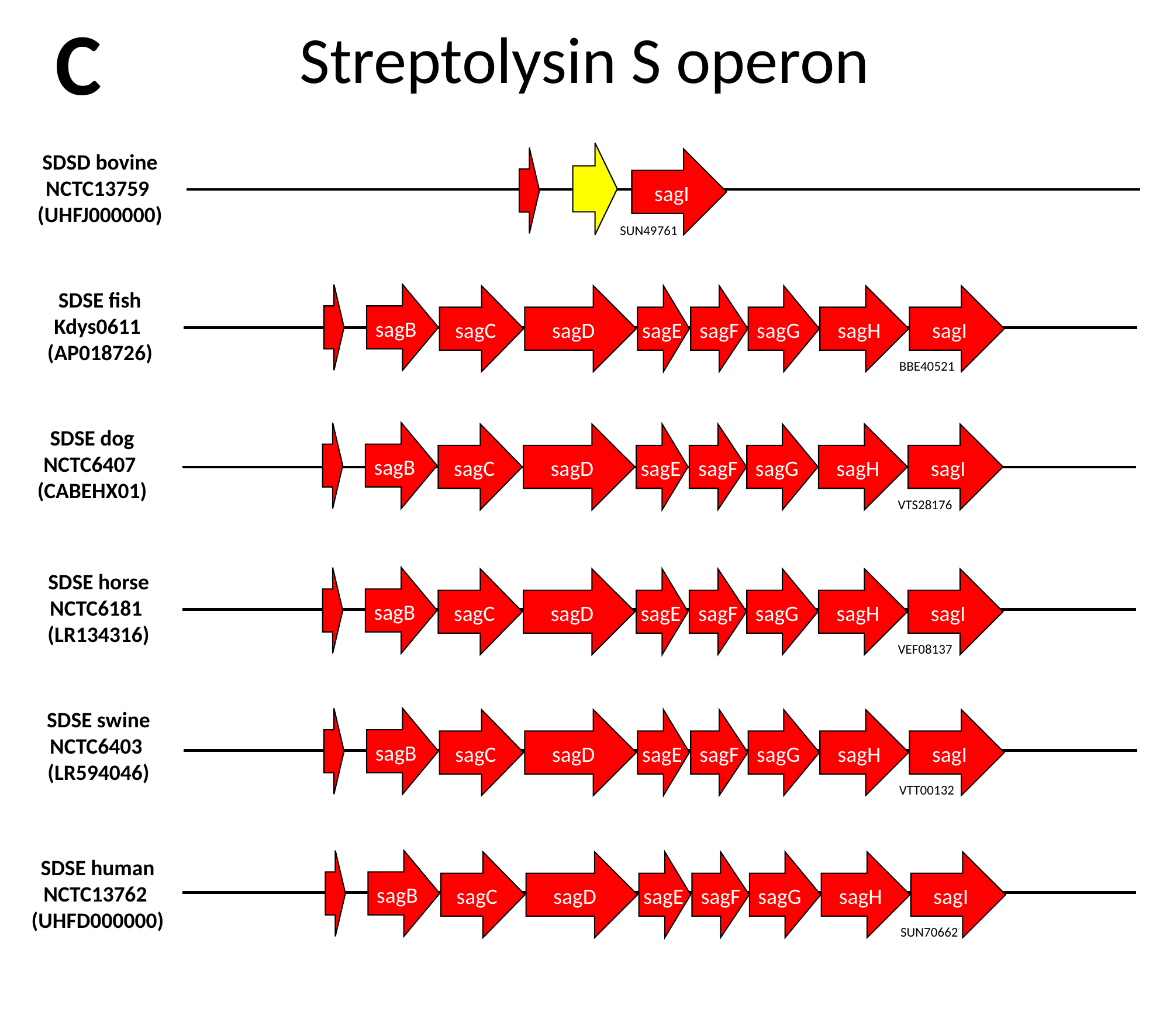

C
Streptolysin S operon
SDSD bovine
NCTC13759
(UHFJ000000)
sagI
SUN49761
SDSE fish
Kdys0611
(AP018726)
sagB
sagG
sagE
sagF
sagH
sagD
sagI
sagC
BBE40521
SDSE dog
NCTC6407
(CABEHX01)
sagB
sagG
sagE
sagF
sagH
sagD
sagI
sagC
VTS28176
SDSE horse
NCTC6181
(LR134316)
sagB
sagG
sagE
sagF
sagH
sagD
sagI
sagC
VEF08137
SDSE swine
NCTC6403
(LR594046)
sagB
sagG
sagE
sagF
sagH
sagD
sagI
sagC
VTT00132
SDSE human
NCTC13762
(UHFD000000)
sagB
sagG
sagE
sagF
sagH
sagD
sagI
sagC
SUN70662

## Slide 5
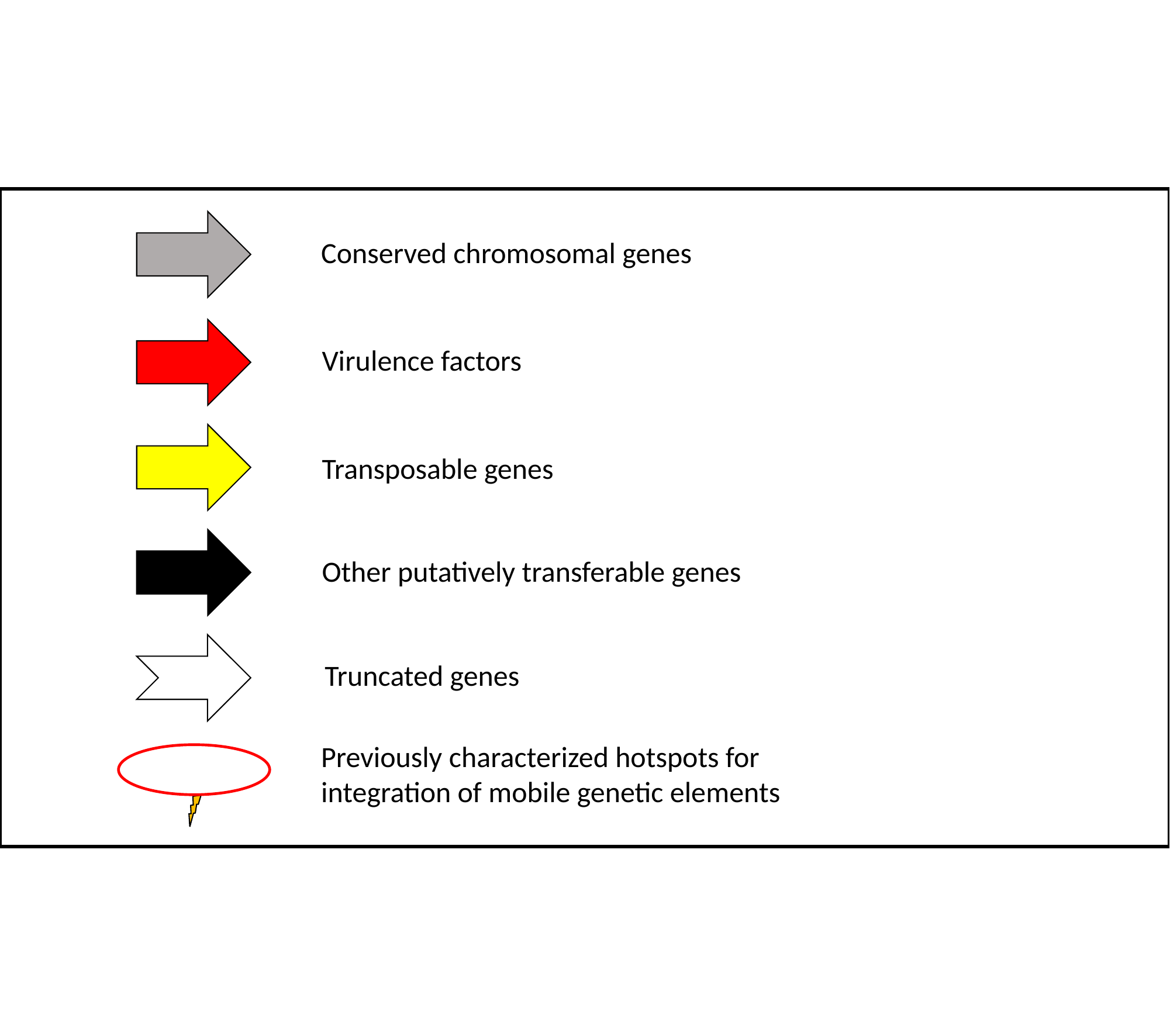

Conserved chromosomal genes
Virulence factors
Transposable genes
Other putatively transferable genes
Truncated genes
Previously characterized hotspots for integration of mobile genetic elements
