# Supplementary material for: Whole genome sequencing reveals possible host species adaptation of Streptococcusdysgalactiae
Source: Sci Rep. 2021 Aug 30;11:17350. doi: 10.1038/s41598-021-96710-z (PMC8405622; doi:10.1038/s41598-021-96710-z)
Supplement: Supplementary file 3 — Supplementary Information 3. [file 41598_2021_96710_MOESM3_ESM.docx]

**Supplementary data**

Figure S1. Pivotal genetic loci distinguishing SDSD and human associated SDSE isolates.

Figure S2. Adaptive evolution in host associated SD lineages.

Table S1. Metadata and genome analysis results for all the strains included in the study, including MLST, M-type and gtfC type.

Table S2. Overview of lineage specific genetic content delineating SDSD and human associated SDSE

Table S3. Mobile genetic elements and associated virulence and resistance genes present in *Streptococcus dysgalactiae* genomes
